# 3D cyclorama for digital unrolling and visualisation of deformed tubes

**DOI:** 10.1038/s41598-021-93184-x

**Published:** 2021-07-19

**Authors:** Charalambos Rossides, Sylvia L. F. Pender, Philipp Schneider

**Affiliations:** 1 Bioengineering Science Research Group, Faculty of Engineering and Physical Sciences, University of Southampton, Southampton, UK; 2 School of Clinical and Experimental Sciences, Faculty of Medicine, University of Southampton, Southampton, UK; 3 Institute for Life Sciences, University of Southampton, Southampton, UK; 4 High-Performance Vision Systems, Center for Vision, Automation & Control, AIT Austrian Institute of Technology, Vienna, Austria

**Keywords:** Cancer imaging, Tumour biomarkers, Engineering, Software, Applied mathematics, Computational science, X-rays, Colorectal cancer

## Abstract

Colonic crypts are tubular glands that multiply through a symmetric branching process called crypt fission. During the early stages of colorectal cancer, the normal fission process is disturbed, leading to asymmetrical branching or budding. The challenging shapes of the budding crypts make it difficult to prepare paraffin sections for conventional histology, resulting in colonic cross sections with crypts that are only partially visible. To study crypt budding in situ and in three dimensions (3D), we employ X-ray micro-computed tomography to image intact colons, and a new method we developed (3D cyclorama) to digitally unroll them. Here, we present, verify and validate our ‘3D cyclorama’ method that digitally unrolls deformed tubes of non-uniform thickness. It employs principles from electrostatics to reform the tube into a series of onion-like surfaces, which are mapped onto planar panoramic views. This enables the study of features extending over several layers of the tube’s depth, demonstrated here by two case studies: (i) microvilli in the human placenta and (ii) 3D-printed adhesive films for drug delivery. Our 3D cyclorama method can provide novel insights into a wide spectrum of applications where digital unrolling or flattening is necessary, including long bones, teeth roots and ancient scrolls.

## Introduction

**Figure 1:**
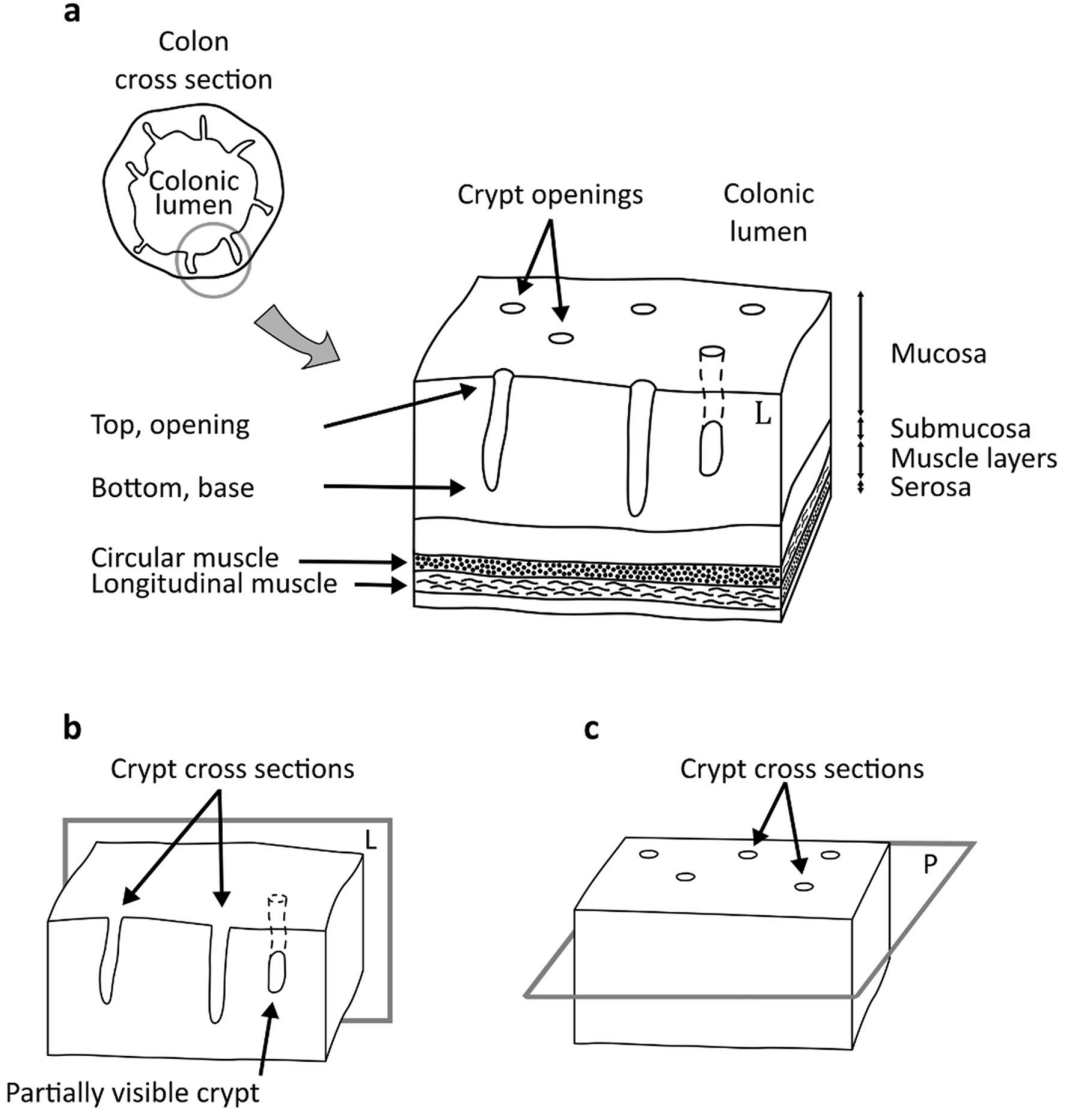
Colonic histology. 3D schematics of the tissue layers in a healthy colon. (**a**) The colon is composed of four main layers: mucosa, submucosa, muscle layers and serosa. The mucosa is the layer closer to the colonic lumen, and it is comprised by a pattern of colonic crypts (tubular glands). (**b**) The crypts have elongated shapes in a cross-sectional view, corresponding to plane ‘L’ (crypts cut longitudinally). Crypts that are cut at an angle by a sectioning plane are only partially visible. (**c**) The crypts have rounded shapes in a cross-sectional view corresponding to plane ‘P’ (perpendicular to the elongated crypts). This figure was created with Inkscape^14^.

In health, crypts or colonic crypts, which are tubular glands in the colon^1^ (see Fig. 1), multiply through a symmetric branching process called ‘crypt fission’. The process begins with a single parent crypt that splits into two identical daughters^2,3^. During the early developmental stages of colorectal cancer (CRC), the fission process is disturbed^2,4,5^. This leads to asymmetrical branching or budding, where the splitting process does not complete, and the daughter crypt remains attached to its parent. Standard practice in CRC research involves the use of animal models, where animals are sacraficed and their colon excised. Small tissue samples of the colon are typically isolated, cut open longitudinally and flattened before being embedded in wax blocks to examine the morphological changes of colonic crypts^6–9^. Paraffin sections of the physically flattened and embedded colons may then be taken parallel to the colonic mucosa^10^ to study the shapes of the crypt cross sections (see Fig. 1). Conversely, when it is necessary to capture the entire crypt on a microscope slide, for instance to study crypt budding, the physical sections of the colon need to be taken along individual crypts, perpendicular to the colonic mucosa and across different tissue layers^11,12^. In the specific case where the morphology of crypt budding is of interest, the challenging shapes of the crypts make it difficult to (routinely) prepare such sections along individual crypts, resulting in colonic cross sections with crypts that are only partially visible (see Fig. 1). Traditionally, this involves turning the murine colon into a Swiss roll, a technique whereby the entire intestine (approximately 6–7 cm in length) is cut longitudinally, laid flat, then rolled into a spiral before chemical fixation and paraffin embedding^13^. After this procedure, the distal colon is then usually located at the centre, whilst the proximal colon at the outside of the roll. Two-dimensional (2D) cross-sectional cut sections of the roll allow for histological studies of morphological abnormalities or any other tissue features over the whole length of the animal colon using conventional light microscopy.

X-ray computed tomography (CT) has been introduced as a three-dimensional (3D) clinical imaging technique in the 1970s. In the decades that followed, improvements in the attainable spatial resolution^15^ have been achieved and X-ray micro-computed tomographic (μCT) volumetric imaging has been developed to non-destructively image small samples (order of cm^3^ and smaller) ex vivo, in 3D, and at exceedingly higher spatial resolutions (order of μm)^16^. Using soft tissue-optimised μCT, the 2D nature of traditional thin-section histology can be expanded to the native 3D world (‘3D X-ray histology’)^17^. As μCT is a non-destructive 3D imaging technique, embedded tissue samples can be imaged in their original shape (deformed tubes), without any structural deformations that come along with physical cutting and/or rolling into Swiss rolls, respectively. Imaging and visualising the whole colonic volume in 3D by μCT is beneficial because the native shape of the colonic crypts is preserved for morphological analysis. Digital unrolling of the imaged colonic volume would facilitate the investigation of tubular samples at different tissue depths, and enable identification of crypt budding.

Apart from colons, volumes that are roughly cylindrical in shape and include features extending radially through the tissue depth, are prominent in biological tissues. Examples are teeth roots made up of layers of annual cementum growth or the tubular shaft of long bones that contain vascular and bone cell networks. We recently applied propagation based phase-contrast X-ray synchrotron radiation CT (SR CT) to non-destructively image fossilised cementum increments in 3D, and recorded lifespans in early mammaliaforms by counting the annual growth increments in tooth-root cementum^18,19^. As cementum increments occasionally split and coalesce, 3D imaging and analysis can be superior to alternative 2D techniques and methods, by avoiding errors in counts of cementum layers based on the analysis of a limited number of 2D cross sections^20^. In bones, the vast bone cell networks formed by osteocytes, the most abundant bone cells, are receiving increasing interest. Imaging and quantifying their 3D structure^21,22^ can enhance the understanding of bone development in health and disease, including osteoporosis and osteoarthritis. 3D imaging approaches for both tooth-root cementum and the osteocyte networks in bone would greatly benefit from a tool to digitally unroll the tubular structure at hand, and represent individual tissue layers with images arranged in a stack. Two further biomedical applications, namely the study of microvilli in the human placenta and 3D-printed pharmaceutical films, are briefly mentioned here as they are discussed in detail as case studies in the ‘Supplementary’ information. Despite the broad range of applications and the utility of a tool for digital volume unrolling, to the best knowledge of the authors, unrolling of deformed objects of non-uniform thickness has not been tackled yet.

Here, we present a new method to digitally unroll the volume of deformed tubes of non-uniform thickness, including but not confined to, colons, teeth roots, long bones, and 3D-printed pharmaceutical films. Our approach reduces the problem of volumetric unrolling of deformed tubes down to the simpler, and already solved problem^23–29^ of mapping 3D surfaces onto planar equivalents. We begin by reformulating the internal volume of the deformed tube into a series of non-intersecting, equidistant, onion-like, 3D surfaces using notions from electrostatics: Virtual electrical charges are applied on the tube’s boundaries, and by deriving the resulting (virtual) electric field lines, we can generate internal 3D surfaces that follow the shape of the deformed tube. We then apply a proof-of-concept approach to map these 3D surfaces onto planar panoramic views that we call ‘3D cyclorama’ (see section ‘Methods: 3D cyclorama design and development’ for a detailed description of the method).

We verified our implementation by testing and quantifying its ability to digitally unroll an in silico phantom we designed for this purpose (see supplementary section ‘S.1. Verification of the 3D cyclorama method’). We then validated our 3D cyclorama method by testing its capability to digitally unroll a murine colon and identify crypt budding during early-stage CRC (see section ‘Validation of the 3D cyclorama method’). Further, two biomedical case studies are presented in the ‘Supplementary’ information on (i) microvilli in ‘S.2 Case study: Human placenta’, and on (ii) 3D-printed pharmaceutical films for drug delivery in ‘S.3 Case study: Pharmaceutical film’. These provide insights into a wide spectrum of potential applications of the presented 3D cyclorama method to unroll deformed cylindrical tubes of non-uniform thickness.

## Methods: 3D cyclorama design and development

### Overview

To the best of the authors’ knowledge, the problem of digital unrolling of deformed tubes has not been solved and addressed in the literature to date. However, the problem of digital unrolling of 3D surfaces has been extensively studied, where predominant applications include straightening wrinkled paper^23–25^ and virtual unrolling of ancient papyri^26–29^. These methods usually attempt to recover text that is written on deformed sheets by fitting 3D surfaces and digitally mapping them onto planar images. Surface meshing (generating a set of interconnected points that represent the surface) and image deformation are also extensively used in computer graphics for image or 3D model representation^30–34^. These methods typically require a set of mapped points (source points mapped to target points) to locally stretch or shrink the image or surface (non-rigid image deformation) so that the source points match up with their targets. When it comes to volumetric image deformation, i.e., flattening of thin sheets with uniform thickness, commercial software such as the plug-in developed by the Konrad-Zuse-Zentrum Berlin (ZIB)^26^ for Amira (Amira 6.5; ZIB/Thermo Fisher Scientific, Germany/USA) are available. However, unrolling volumetric deformed tubes or scrolls of non-uniform thickness adds another level of complexity. Our approach to this problem is to simplify the geometry by reformulating the deformed tube of non-uniform thickness into a series of non-intersecting, equidistant, onion-like 3D surfaces, which may then be mapped onto 2D (planar) equivalents via any of the existing methods.

**Figure 2:**
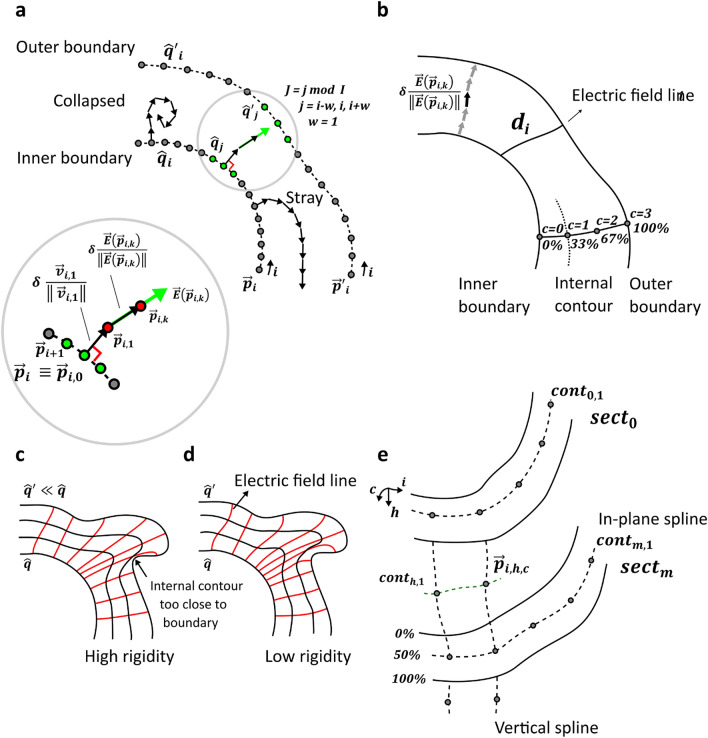
Generation of 3D cycloramas through electrostatic fields. (**a**) Boundary contours are defined on the deformed tube boundaries as sequences of charged points $\vec{p}_{i}$ and $\vec{p}'_{i}$. Each electric field line is built up incrementally starting at point $\vec{p}_{i} \equiv \vec{p}_{{i,0}}$. The first increment $\delta \cdot \frac{{\vec{v}_{{i,1}} }}{{ \lVert \vec{v}_{{i,1}} \rVert }}$ is normal to the inner boundary. Each subsequent increment $\delta \cdot \frac{{\vec{E}\left( {\vec{p}_{{i,k}} } \right)}}{{ \left\lVert \vec{E}\left( {\vec{p}_{{i,k}} } \right) \right\rVert }}$ is defined as the direction of the electric field scaled by $\delta$ (electric field line segment size), computed at the tip of the previous increment $\delta \cdot \frac{{\vec{E}\left( {\vec{p}_{{i,k - 1}} } \right)}}{{ \left\lVert \vec{E}\left( {\vec{p}_{{i,k - 1}} } \right) \right\rVert}},$ considering charges on both boundaries in a user-defined window (green points ). This process might fail to connect point $\vec{p}_{i}$ to the outer boundary, resulting in ‘collapsed*’* or ‘stray*’* lines, which are identified and removed in our cyclorama method. (**b**) The local thickness is defined as the length $d_{i}$ of field line *i*. Intermediate contours $cont_{{h,c}}$ are defined on a total of $C$ relative depth levels $\frac{c}{{C - 1}} \cdot 100\%$ ($c = 0,1, \ldots ,C - 1$). (**c**) Stiff field lines (high *rigidity*) do not properly follow the curvature of the outer boundary. (**d**) Lower *rigidity* results in more flexible field lines, pushing the intermediate contours away from the boundaries. (**e**) The process shown in a-d is performed on every *m*th section of the image stack. The remaining contours $cont_{{h,c}}$ on sections $sect_{h}$ ($h \ne 0,m,2m, \ldots$) are generated by fitting vertical cubic splines. This figure was created with Inkscape^14^.

**Figure 3:**
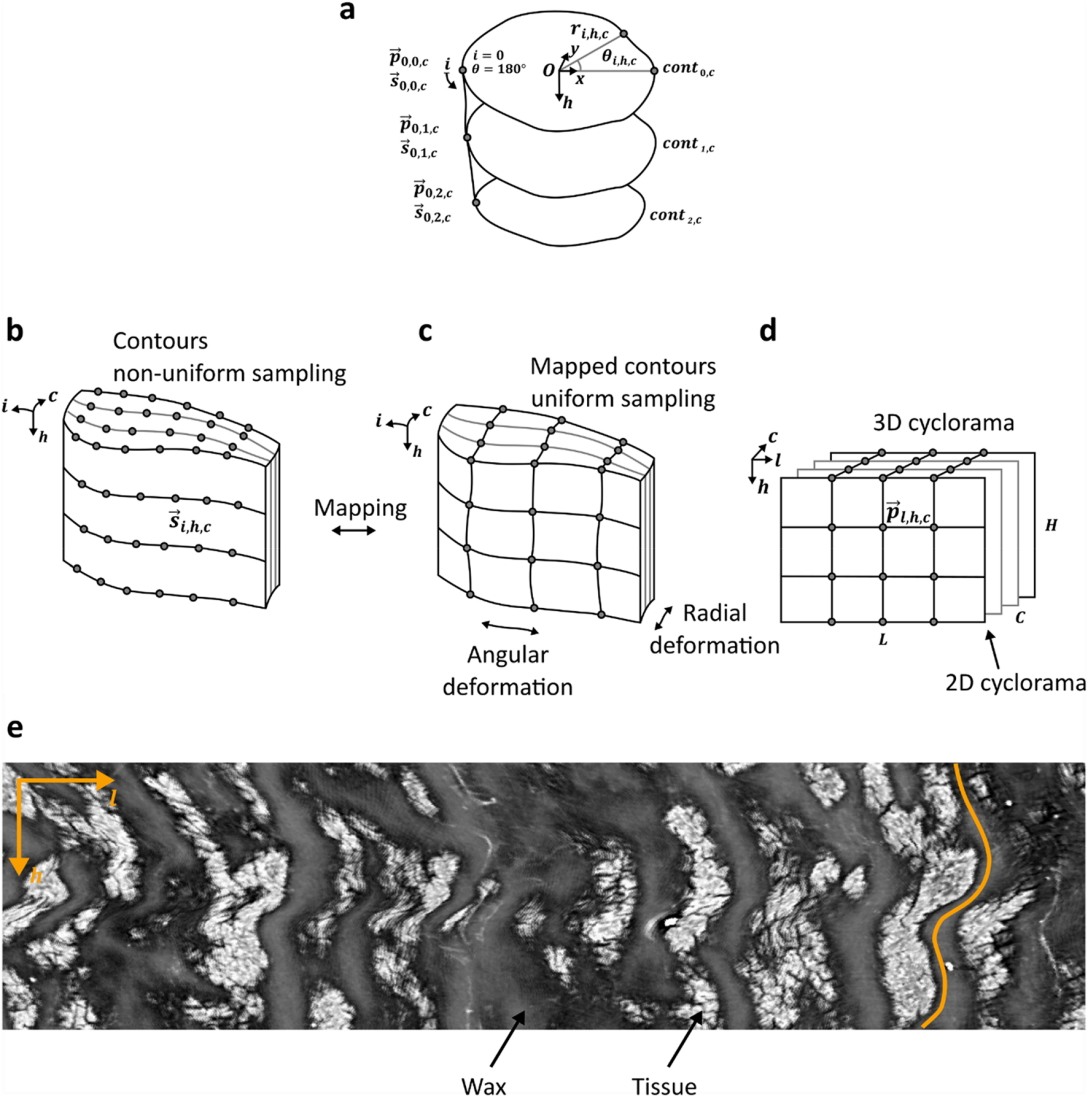
Linear mapping of 3D re-slicing surfaces. (**a**) The re-slicing surface edge at depth level *c* is defined by selecting the first point $\vec{s}_{{i,h,c}} = \vec{s}_{{0,h,c}} = \left( {r_{{0,h,c}} , 180^\circ } \right)$. (**b**) Initial re-slicing surfaces are represented as unstructured contours. (**c**) The points in the unstructured contours in **b** are mapped onto the structured grid in **c**. (**d**) The structured grid shown in **c** is used to probe the 3D image data and create the 3D cyclorama. (**e**) A section of a 2D cyclorama at 0% relative depth level ($c = 0$: inner boundary) of a healthy murine colon embedded in paraffin wax, which partially probes volumes from the background (paraffin wax) and the sample (tissue). Vertically curved patterns are visible, as revealed by the orange outline of the tissue. This is a consequence of local image deformations (stretching/contraction), which results in anisotropic pixel sizes. (**a-d**) Sketches **a-d** were created with Inkscape^14^.

We first define the tube volume using the inner and outer boundary of the tube in 3D. Given an image stack depicting the deformed cylindrical tube, these boundaries are defined as a collection of inner and outer boundary contours drawn manually on 2D image slices. We then virtually charge the sample boundaries with (virtual) electrical charges, and simulate the field lines of the resulting electrostatic field (see Fig. 2a). This allows us to define intermediate or internal contours that are non-intersecting and follow the shape of the sample. Each internal contour is defined at a certain percentage of the local thickness of the deformed tube (see Fig. 2b) so that each internal contour corresponds to a relative local thickness. The internal contours are used to create internal (onion-like) re-slicing surfaces. This is the point where the problem is simplified to the already solved problem of mapping re-slicing surfaces onto 2D equivalents: Several approaches exist to solve this problem, as mentioned earlier (see also section ‘Discussion’), with varying degrees of complexity. Here, we adopt a simple method to non-rigidly map the re-slicing surfaces onto rectangular 2D surfaces. The 2D (planar) surfaces are stacked together to provide panoramic views at different depths of the tubular volume, which we call ‘3D cyclorama’ (see Fig. 3d).

We implemented and tested the 3D cyclorama method as a Jython script in Fiji^35^ (ImageJ version 2.0.0-rc-68/1.52 g, Java 1.8.0_66), a scientific image processing software package written in Java that is distributed as an extended version of ImageJ^36,37^. Jython is an implementation of the Python programming language that runs on Java platforms and is provided as one of the available scripting languages in Fiji. In the following, we describe in detail the implementation and validation of our 3D cyclorama method.

### Meshing: generation of internal contours and re-slicing surfaces

A sampling grid (set of onion-like surfaces) within the deformed tube is generated as a set of intermediate or internal contours, corresponding to incremental relative local depth levels (0% to 100%) as follows: Given is a stack of images depicting 2D cross sections of a vertically aligned deformed tube, for instance provided as individual reconstructed CT slices at different heights of the sample. Thus, each slice represents a deformed 2D annulus that is defined by its inner and outer boundaries. The boundaries are subsequently used to interpolate the enclosed cross-sectional area and describe the internal contours at different relative local thickness levels.

We define the local thickness by employing notions from physics, namely electrostatics. Firstly, we assume that the inner and outer boundaries of the cross sections are loaded by opposite electrical charges. We calculate the (virtual) 2D electric field lines within the enclosed space described by those boundaries as shown in Fig. 2. The theory governing electrostatic fields^38^ ensures that electric field lines never cross and that they never form closed loops (they always start at one boundary and cross the tube to reach the other boundary). Moreover, the electric field lines are always perpendicular to the charged boundaries. These properties make the electric field lines an ideal tool for the definition of the relative local 2D thickness, which we define here as the length $d_{i}$ of the respective electric field line *i*, spanning the inner and outer boundary of the tube’s cross section (see Fig. 2b).

The electric field $\vec{E}$ at point $\vec{p}$ is defined as the sum of the contributions from points $\vec{p}_{j}$ in space, each charged with an electric charge $q_{j}$:

1 $$\vec{E}\left( {\vec{p}} \right) = \mathop \sum \limits_{{j = 1}}^{N} \frac{{q_{j} }}{{4\pi \varepsilon _{o} }}\frac{{\left( {\vec{p} - \vec{p}_{j} } \right)}}{{\left\| {\vec{p} - \vec{p}_{j} } \right\|^{3} }},$$

where $\varepsilon _{o}$ is the electric constant, $j = 1 \ldots N$ is the set of contributing charges, and || || denotes the Euclidean distance. Due to the computational costs involved to calculate the exact electric field at all possible positions in space, Eq. (1) was not implemented directly here, but the following computational approach has been developed to approximate the electric field lines:

1. First, the boundary contours are defined as sequences of equidistant points $\vec{p}_{i}$ and $\vec{p}'_{i}$ ($i = 0,1, \ldots, I - 1$) for the inner and outer boundary, respectively, with the total number of points $I$ being a (positive) integer given by the user. These points define the positions of the electrical charges that are used for the calculation of the field lines. Each point on the contour can be identified by an index $i$ that is used to select the contributing charges as discussed next (see Fig. 2). Note that for tubes the boundary (and internal) contours are closed, and hence the locations of the starting points $\vec{p}_{{i = 0}}$ and $\vec{p}'_{{i = 0}}$ are arbitrary. However, it is important for the generation of cycloramas (see section ‘Mapping: Generation of 3D cycloramas’) that the sequences of points on the internal and external boundary are paired, i.e., points with index $i$ on the two opposing boundary contours are fairly close to each other. This will be ensured via appropriate selection of the starting points.

2. Each electric field line $i$ is built up incrementally, by starting at the location of charge ${q}_{i}$ (point $\vec{p}_{i} \equiv \vec{p}_{{i,0}}$ on the inner boundary contour) and computing the electric field at points $\vec{p}_{{i,k}}$. Due to the term $\left\| {\vec{p} - \vec{p}_{j} } \right\|$ in the denominator of Eq. (1) singularities may arise when point $\vec{p}$ is very close to the charged points $\vec{p}_{j}$, as this would result in division by zero. To avoid this problem, the first calculation of the electric field is bypassed and replaced by progressing along the direction of the vector $\vec{v}_{{i,1}}$, which is perpendicular to the boundary contour at the location $\vec{p}_{{i,0}}$ of charge $q_{i}$, pointing inwards the annulus (see Fig. 2a). The vector $\vec{v}_{{i,1}}$ is calculated as the vector perpendicular to the tangent vector $\vec{u}_{i} = \left( {u_{{i,x}}, u_{{i,y}} } \right): = \vec{p}_{{i + 1,0}} - \vec{p}_{{i,0}}$ as follows:

2 $$\vec{v}_{{i,1}} : = \pm \left( {u_{{i,y}} , - u_{{i,x}} } \right).$$

The first step for each field line *i* is calculated by progressing in a straight line along the direction of the vector $\vec{v}_{{i,1}}$ for a user-defined length $\delta$ (‘electric field line segment size’ in number of pixels):

3 $$\vec{p}_{{i,1}} = \vec{p}_{{i,0}} + \delta \cdot \frac{{\vec{v}_{{i,1}} }}{{\left\| {\vec{v}_{{i,1}} } \right\|}}.$$

Note that Eq. (2) has two solutions (±), one points towards the annulus and the other points away from the annulus. The correct solution is chosen by selecting the resulting point $\vec{p}_{{i,1}}$ that lies within the annulus. Since the inner and outer boundaries are closed lines, the annulus may be defined as the enclosed region. In ImageJ the contours are defined as regions of interest (ROIs) and the annulus is computed as their set difference (XOR function).

3. As the magnitude of the electric field drops with the second power of the distance from the contributing charges (see Eq. (1)), we approximate the electric field at point $\vec{p}_{{i,k}}$ by only considering charges that are close to point $\vec{p}_{{i,k}}$. The contributing charges are selected as the sequences of points on the two boundary contours within a user-defined ‘electric field search window’ around index *i* (see Fig. 2a):

4 $$J: = j\;mod\;I,\;j = i - w, \ldots, i, \ldots ,i + w,\quad w \in \mathbb{N},$$

where *w* is a user-defined integer that defines the window size. The modulo operator $mod$ enables ‘closing’ the contours by selecting points at the two ends of the sequence $j = i - w, \ldots, i, \ldots, i + w$, $w \in \mathbb{N}$, when *i* is too small $(i < w)$ or too large $(i > I - w)$. The electric field line *i* is built upon the first computed point $\vec{p}_{{i,1}} = \vec{p}_{{i,0}} + \delta \cdot \frac{{\vec{v}_{{i,1}} }}{{\left\| {\vec{v}_{{i,1}} } \right\|}}$ as a sequence of straight electric field line segments of length $\delta$ (electric field line segment size):

5 $$\vec{p}_{{i,k}} = \vec{p}_{{i,k - 1}} + \delta \cdot \frac{{\vec{E}\left( {\vec{p}_{{i,k}} } \right)}}{{\left\| {\vec{E}\left( {\vec{p}_{{i,k}} } \right)} \right\|}},$$

where

6 $$\vec{E}\left( {\vec{p}_{{i,k}} } \right) = \mathop \sum \limits_{{j \in J}} \hat{q}_{j} \frac{{\left( {\vec{p}_{{i,k}} - \vec{p}_{{j,0}} } \right)}}{{\left\| {\vec{p}_{{i,k}} - \vec{p}_{{j,0}} } \right\|^{3} }} + \mathop \sum \limits_{{j \in J}} \hat{q}'_{j} \frac{{\left( {\vec{p}_{{i,k}} - \vec{p}'_{{j,0}} } \right)}}{{\left\| {\vec{p}_{{i,k}} - \vec{p}'_{{j,0}} } \right\|^{3} }},$$

and integer $k = 2 \ldots K$ is the index of the electric field line at point $\vec{p}_{{i,k}}$, points $\vec{p}_{{j,0}}$ are the locations of the contributing charges $\hat{q}_{j}$ on the inner boundary contour, points $\vec{p}'_{{j,0}}$ are the locations of the contributing charges $\hat{q}'_{j}$ on the outer boundary contour (green points in Fig. 2a), where $\hat{q}_{j}$ and $\hat{q}'_{j}$ are arbitrary positive and negative electric charges, respectively. This process is repeated until either the electric field line reaches the opposite boundary, or a user-defined maximum number of steps $K_{{max}}$ has been reached. Whether the field line has reached the outer boundary is checked by verifying if $\vec{p}_{{i,k}}$ lies within the annulus or on/beyond the outer boundary contour. Note that Eq. (4) defines a window of $2w + 1$ points on the inner and outer boundary contours that slides with the index *i* of the respective field line. This selection includes neighbouring points confined within the boundary contours, which are not necessarily the closest points to $\vec{p}_{{i,k}}$ in terms of in-plane Euclidean distance, as required by Eq. (1). This might lead the algorithm to fail with ‘stray’ or ‘collapsed’ field lines due to inaccurate approximation of the electric field, as discussed next. To improve the estimation, one can increase *w* at the expense of computational time.

A beneficial bi-product of using electric fields for the definition of the internal contours is the control it provides over the curvature of the electric field lines. This may be tuned via appropriate selection of the relative amplitude of the charges on the boundaries of the tube, a parameter we may call ‘rigidity’ (see Fig. 2c-d). In practice, this is achieved by defining $\hat{q}_{j} = 1$ for all charges on the inner boundary and $\left| {\hat{q}'_{j} } \right| = rigidity$ (user-defined positive, real value) for the charges on the outer boundary. This essentially weights the contributions to the electric field defined in Eq. (6), provided by the charges on the outer boundary $(\hat{q}'_{j} )$ vs. the contributions of the charges on the inner boundary $(\hat{q}_{j} )$. Tuning the rigidity results in straighter or more curved electric field lines, thus providing flexibility to capture irregular tubular shapes that are heavily deformed (see Fig. 2d).

The computational approach to iteratively approximate the electric field lines described above reduces the computational cost to calculate the exact value of the electric field at all possible positions in space, as per Eq. (1). This approximation, involving a (i) discrete, user-defined electric field line segment size $\delta$ for progressing through the annulus, (ii) a user-defined electric field search window ($J = j\,mod\,I, j = i - w, \ldots,i, \ldots ,i + w$, $w \in \mathbb{N}$) encompassing a limited number of contributing charges, and (iii) manual tuning of the relative electric charge magnitudes or rigidity, might provide ‘stray’ or ‘collapsed’ electric field lines that do not reach the opposite boundary (see Fig. 2a). Such field lines are identified and removed as follows: Stray lines are defined/identified as exceedingly long lines, and they are rejected by introducing a maximum number of iterations for the computation of each electric field line: $K \le K_{{max}}$. Collapsed field lines are defined as lines that do not progress far from the initial point $\vec{p}_{{i,0}}$, and they are identified as the lines with variance $V_{i}$ less than a user-defined minimum electric field line variance $V_{{min}}$:

7 $$V_{i} = \frac{1}{K}\mathop \sum \limits_{{k = 0}}^{K} \left( {\vec{p}_{{i,k}} - \overrightarrow {{\overline{{p_{i} }} }} } \right)^{T}\cdot \left( {\vec{p}_{{i,k}} - \overrightarrow {{\overline{{p_{i} }} }} } \right),$$

where

8 $$\overrightarrow {{\overline{{p_{i} }} }} = \frac{1}{K}\mathop \sum \limits_{{k = 0}}^{K} \vec{p}_{{i,k}} .$$

Each electric field line *i* is then represented by a cubic spline fitted through all points $\vec{p}_{{i,k}}$ ($k = 0 \ldots K$). Note that once the electric field lines are transformed form a collection of points $\vec{p}_{{i,k}}$ to cubic splines, the notation $\vec{p}_{{i,k}}$ is hence abandoned and the cubic splines are resampled as follows: Firstly, the local thickness $d_{i}$ at $\vec{p}_{i}$ is defined as the length (in number of pixels) of the electric field line *i*. Internal contours are then created at a total of *C* (user-defined) depth levels at incremental percentages (0%-100%) of each individual electric field line’s length $d_{i}$ (see Fig. 2b): Relative depth levels are defined as $\frac{c}{{C - 1}} \cdot 100\%$ and absolute depth levels as $\frac{c}{{C - 1}} \cdot d_{i}$ ($c = 0,1, \ldots ,C - 1$) with *c*, *C* being positive integers. Explicitly, for $C = 3:\frac{0}{2} \cdot 100\% = 0\%$ (inner boundary), $\frac{1}{2} \cdot 100\% = 50\%$, $\frac{2}{2} \cdot 100\% = 100\%$ (outer boundary). See Fig. 2b for an example of four (*C* = 4) different relative depth levels (0%, 33%, 67%, 100%). The number of relative depth levels *C* determines the number of equidistant, non-overlapping contours (including the internal boundary at 0% and the external boundary at 100% relative depth level), which can be chosen arbitrarily. Each contour $cont_{{h,c}}$ is defined by fitting a cubic (in-plane) spline at height level *h* through all points on the electric field lines *i* that correspond to depth level *c* or absolute depth levels $\frac{c}{{C - 1}} \cdot d_{i}$. As some of the electric field lines may have been discarded (stray or collapsed field lines) the resulting internal contours might consist of fewer points than the boundary contours. To obtain a uniform representation, each cubic spline representing one specific contour $cont_{{h,c}}$ is resampled with a total of *I* samples.

As the approximation of the electric field lines is the most computationally intensive part of our method to generate 3D cycloramas, it is only applied on every *m*th slice or 2D section of the 3D image stack (positive integer *m*: interpolation interval) that depicts the deformed tube, resulting in contours $cont_{{h,c}}$ on sections $sect_{h}$ ($h = 0,m,2m, \ldots$ and $h \le H - 1$ with *H* the total number of slices) at depth level *c* as shown in Fig. 2e. To generate the contours $cont_{{h,c}}$ on the remaining sections $sect_{h}$ ($h \ne 0,m,2m, \ldots$), the contours of the selected 2D cross sections are then interpolated across all sections of the 3D image stack. In detail, vertical cubic splines are fitted on corresponding points, i.e., the *i*th spline is created by selecting the *i*th point on each contour for each depth level *c* (see Fig. 2e). Eventually $cont_{{h,c}}$ is defined as the set of all points at cross section *h* and depth level *c*: $cont_{{h,c}} = \{ \vec{p}_{{i,h,c}} \}$.

Once contours have been defined at all depth levels *c* and for all 2D cross sections *h*, each 3D re-slicing or an onion-like surface at relative depth level $\frac{c}{{C - 1}} \cdot 100\%$ is defined as the set of all 2D contours at depth level *c*. The basic components are then ready to generate internal 2D panoramic views of the deformed cylindrical sample and 3D cycloramas as explained in the next section. At this point, the problem of volumetric unrolling of deformed tubes has been reduced to the simpler and already solved problem of manifold embedding or mapping 3D surfaces onto 2D equivalents, as discussed next.

### Mapping: generation of 3D cycloramas

To generate 2D panoramic views at different relative depth levels $\frac{c}{{C - 1}} \cdot 100\%$ of the deformed tube, the 3D re-slicing surfaces need to be mapped onto 2D equivalents (2D cycloramas). The problem has been widely studied in the literature, and several interesting approaches are discussed in section ‘Discussion’. Here, we adopt a simple and basic approach as a proof of concept. Mapping is performed on a contour-by-contour basis, where the boundary and internal contours need to be mapped onto rectangular grids. When stacked together, these 2D panoramic views become a 3D cyclorama, which displays the boundaries and particularly the internal structure of the sample at different depth levels (see Fig. 3).

Initially, the length of all 2D panoramic views or 2D cycloramas is fixed at reference length *L*, defined by the longest contour in the volume: $L: = \mathop {\max }\limits_{{h = 0\; \ldots \;H - 1}} \left( {\mathop {\max }\limits_{{c = 0\; \ldots \;C - 1}} L_{{h,c}} } \right)$, with integer $L_{{h,c}}$ being the length of contour $cont_{{h,c}}$ of section $sect_{h}$ at relative depth level *c* (see Figs. 2e and 3b) in number of pixels (see Fig. 3d). The longest contour thus defines the length *L* of the 3D cyclorama, while all other contours ($L_{{h,c}} \le L$) are dilated to match up with the longest contour. On this account, the resulting (non-uniform) pixel size of the 3D cyclorama will be equal (for the longest contour) or larger (all other contours) when compared to the pixel size of the original image data.

We then open the closed contours into sequences, by resampling each of the contour splines so that contour $cont_{{h,c}}$ is (densely) represented by a total of $L_{{h,c}}$ points. Then, the first point $\vec{p}_{{0,h,c}}$ (point with index $i = 0$), is arbitrarily but consistently selected across all contours $cont_{{h,c}}$ as follows (see Fig. 3a): We can express the points of each contour $cont_{{h,c}}$ that are initially defined in Cartesian coordinates $\vec{p}_{{i,h,c}} = \left( {p_{{i,h,c_{x}}} , p_{{i,h,c_{y}}} } \right)$ in polar coordinates:

9 $$\vec{s}_{{i,h,c}} = \left( {r_{{i,h,c}} ,\theta _{{i,h,c}} } \right),\quad i = 0 \ldots L_{{h,c}} - 1,$$

with $O = \left( {o_{x} ,o_{y} } \right)$ an arbitrary rotation axis inside the tube, $r_{{i,h,c}}$ the Euclidean distance from the rotation axis, and $\theta _{{i,h,c}} \in \left[ {0 \ldots 2\pi } \right)$ the azimuthal angle in radians. The first point $\vec{p}_{{0,h,c}}$ or $\vec{s}_{{0,h,c}}$ is chosen as the point among all points on contour $cont_{{h,c}}$ with $\theta _{{i,h,c}}$ closest to $\pi$ radians or 180 degrees (see Fig. 3a).

Mapping of the boundaries and internal contours onto 2D cycloramas (with a fixed length *L*) is performed by uniformly sampling each boundary and internal contour with the same number of points as the longest/reference contour (see Fig. 3c). In other words, all contours except for the longest/reference contour are resampled once again, so that contour $cont_{{h,c}}$ is represented by a total of *L* points, effectively stretching each contour to fit the longest contour in the volume. This ‘linear’ mapping avoids collapsing of points and contraction. It results in a uniform pixel size along individual lines (horizontal or *l*-dimension in Fig. 3e) of the cyclorama image but not necessarily the same pixel size across all lines (vertical or *h*-dimension in Fig. 3e), since each contour is stretched individually. This effectively spreads the image deformation across the entire image, resulting in wavy but continuous cycloramas as shown in Fig. 3e. Note that any mapping approach tries to spread the inevitable image deformation throughout the image, typically by applying a mapping that is optimal in some respect. Alternative approaches are discussed in section ‘Discussion’. The mapping process we implemented defines which index *i* corresponds to each index *l* in the stretched contour as follows:

10 $$i \leftrightarrow l, i \in \left\{ {0 \ldots L_{{h,c}} - 1} \right\},\quad l = 0 \ldots L - 1.$$

These indices are then used to redefine the contours by selecting the points $$\vec{s}_{{i,h,c}}$$ that correspond to each point $$\vec{p}_{{l,h,c}} = \left( {l,h,c} \right)$$ in the 3D cyclorama (see Fig. 3b-d). The mapping between the 3D surface, defined by all points with fixed depth level $$\overset{\lower0.5em\hbox{$\smash{\scriptscriptstyle\smile}$}}{c}$$, onto points on the 2D cyclorama plane is then defined as:

11 $$\vec{s}_{{i,h,\overset{\lower0.5em\hbox{$\smash{\scriptscriptstyle\smile}$}}{c} }} \leftrightarrow \vec{p}_{{l,h,\overset{\lower0.5em\hbox{$\smash{\scriptscriptstyle\smile}$}}{c} }} .$$

One (planar) 2D cyclorama per depth level $$\overset{\lower0.5em\hbox{$\smash{\scriptscriptstyle\smile}$}}{c}$$ is created by probing the 3D image data volume at all points $$\vec{s}_{{i,h,\overset{\lower0.5em\hbox{$\smash{\scriptscriptstyle\smile}$}}{c} }}$$ (see Fig. 3b-d). Note that all 2D cycloramas, which correspond to a specific relative depth level, are of equal size (equal length and height). This allows to create a stack of 2D cycloramas from 0% (internal boundary) to 100% (external boundary) of the tube’s relative depth level (3D cyclorama), which is essentially an unrolled version of the deformed tube. This digital unrolling (onto a 3D cyclorama) has been achieved by introducing image deformations in two directions: Radially, through the definition of the relative depth levels using the electric field lines, and along contours, by stretching the contours (see Fig. 3c).

## Validation of the 3D cyclorama method

Here we test the utility of our method for the study of crypt budding in early-stage colorectal cancer (CRC). We employ a well-established murine model, and prepare cylindrical formalin-fixed paraffin-embedded (FFPE) colon samples with early-stage CRC. A sample preparation method is employed, where the original tubular shape of the colons is preserved. We then apply non-destructive 3D imaging using X-ray phase-contrast μCT, followed by digital unrolling using our 3D cyclorama method. This enables us to easily identify crypt budding in the 3D cycloramas and perform image segmentation of the budding crypts on the μCT data, where the original shape and orientation of the crypts is preserved. This approach allows for accurate quantification of the 3D shape of the crypts using μCT and subsequent functional studies using traditional paraffin sectioning and histology methods.

### Validation methods

#### Animal model

An azoxymethane and dextran sodium sulphate (AOM/DSS) murine model was used to produce a colon sample with early stage CRC. The model uses AOM, which is a procarcinogen that inflicts DNA damage before it is excreted into the bile and taken up by the colonic epithelium^39^, and DSS, which is a polysaccharide that inflicts epithelial damage in the colon and inflammatory response^40^. The specific protocol applied here was adapted from Neufert et al.^41^ and Parang et al.^42^ as follows: A C57BL/6J male mouse that was 8 weeks old at the beginning of the experiment was treated with one intraperitoneal injection of 10 mg/kg AOM solution on day 1. Next, starting on day 3, the mouse was treated with 2% weight/volume DSS solution in sterile water for 7 days, followed by 14 days of sterile water provided ad libitum, via a mouse water drinking bottle (inflammation and healing phases, respectively). One healthy (untreated) and one AOM/DSS-treated mouse were culled via schedule 1 on day 24 and the distal colons were excised. In order to preserve the original shape of the colonic tissue, the lumen was filled with fixative before the two ends were sealed with sutures. The resulting sample looks like a ‘sausage’^13^. The samples were fixed in 10% neutral buffered formaldehyde for 2 days, before 10 mm-long tubular pieces were embedded in paraffin wax using a plastic drinking straw as a mould to create cylindrical paraffin wax blocks.

The animals were bred and maintained in the Biomedical Research Facility, University of Southampton, Southampton, UK, in accordance with the United Kingdom Animals (Scientific Procedures) Act 1986. This study has received ethical approval from the Animal Welfare and Ethical Review Body (AWERB) of the University of Southampton, Southampton, UK, and was operated under an existing Home Office animal (scientific) procedures project licence. The regulatory protocols were performed according to the animal research reporting of in vivo experiments (ARRIVE) guidelines^43^. The animals were kept at constant temperature of 22 ± 2 °C with food and filtered water available ad libitum, and were sacrificed under the Schedule 1 procedure.

#### X-ray CT imaging

**Figure 4:**
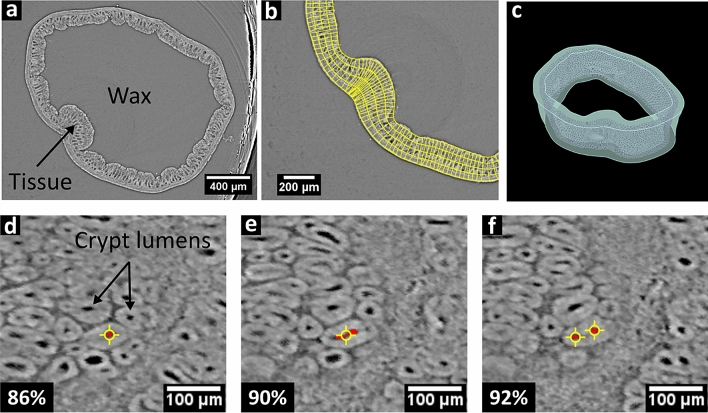
3D cycloramas for the study of murine colons. (**a-c**) A healthy murine colon embedded in paraffin wax. (**a**) A SR CT section of the intact colon. (**b**) Virtual electric field lines and the resulting contours ($C = 5$ instead of the total $C = 30$ depth levels are shown here for visual clarity). (**c**) CT grey values are shown at 50% relative depth level, while boundary surfaces are shown transparent. (**d-f**) Unrolled CT stack of a murine colon with early stage colorectal cancer. The 3D cyclorama has a total of $C = 50$ slices or depth levels, where sequential slices at relative depth levels of 86%, 90% and 92% correspond to slices 42/49, 44/49 and 45/49, respectively. Crypt budding is revealed when a single colonic cross section, shown here in red, divides from one to several branches when moving from one slice to the next at a different colonic tissue depth, i.e., moving from **e** to **f**. Yellow crosses show points created inside individual budding crypts on each cyclorama slice. (**c**) Image **c** was created using Mayavi^46^.

Both samples were imaged using synchrotron light-based X-ray CT at beamline I13-2 of the Diamond Light Source, Didcot, UK, at an energy of 25 keV, exposure time of 85 ms per X-ray projection for 2501 projections, an (isotropic) voxel size of 2.2 μm and an X-ray propagation distance (sample-to-detector distance) of 230 mm. Phase retrieval was performed using the Paganin algorithm^44^, prior to standard filtered back projection (FBP)^15^ for CT reconstruction using an in-house implementation (Savu version 2.2)^45^. From each of the resulting CT stacks (see Fig. 4a), a subset of 301 reconstructed CT slices (height of 0.66 mm) were extracted, each 1841 × 1841 pixels^2^ or 4.1 × 4.1 mm^2^ in size.

#### Image processing and visualisation

In order to digitally unroll the samples using our 3D cyclorama method the boundaries of their tubular volume were manually defined as follows: For the healthy sample all tissue layers were selected via manual definition of the boundary contours at the boundaries between the tissue and the paraffin wax (see Fig. 4a-b). For the sample of the AOM/DSS-treated mouse the mucosal layer was selected via manual definition of the boundary contours, in order to isolate the crypts. Both samples were unrolled with $I = 200$ point-long contours, electric field search windows of 31 points ($w = 15$), electric field line segment sizes of $\delta = 2$ pixels, minimum electric field line variance $V_{{min}} = 50$, rigidity equal to 1, interpolation interval $m = 20$ and rotation axis origin $O = \left( {1080,780} \right)$. For the healthy sample a total of $C = 30$ depth levels and a maximum number of steps $K_{{max}} = 75$ were selected. The resulting 3D cyclorama’s length was $L = 2582$ pixels, the height $H = 301$ pixels, and the depth $C = 30$ slices. For the AOM/DSS-treated sample a total of $C = 50$ depth levels for increased spatial resolution in the *c*-direction and a maximum number of steps $K_{{max}} = 50$ were selected (see Fig. 2d). The resulting 3D cyclorama had a length of $L = 2530$ pixels, height $H = 301$ pixels, and depth $C = 50$ slices. The unrolling process, including the processing and writing the results to hard disk, took less than five minutes for each sample (laptop computer with Intel Core i5-6300U @ 2.5 GHz, 2 cores, 4 logical processors, and 16 GB of RAM).

We firstly identified crypt budding on the 3D cyclorama, and subsequently performed image segmentation and quantification on the original 3D CT stack, and not on the reformed 3D cyclorama, as follows: After unrolling, sequential slices of the 3D cyclorama depict cross sections of the crypts at different depths of the colonic tissue. We traced individual crypts through the colonic tissue layers at different depths, i.e., through individual layers of the 3D cyclorama. We then identified non-budding crypts as those with a single cross section that did not divide on any of the cyclorama layers representing different colonic tissue depths. Budding crypts were identified by pinpointing those locations where a single colonic cross section divided from one to multiple cross sections. We then manually created a point (‘Point tool’ in Fiji) inside each of the crypts on each cyclorama layer. Hence, each crypt was represented by a set of points in 3D defined on the individual cyclorama layers (Fig. 4d-f).

**Figure 5:**
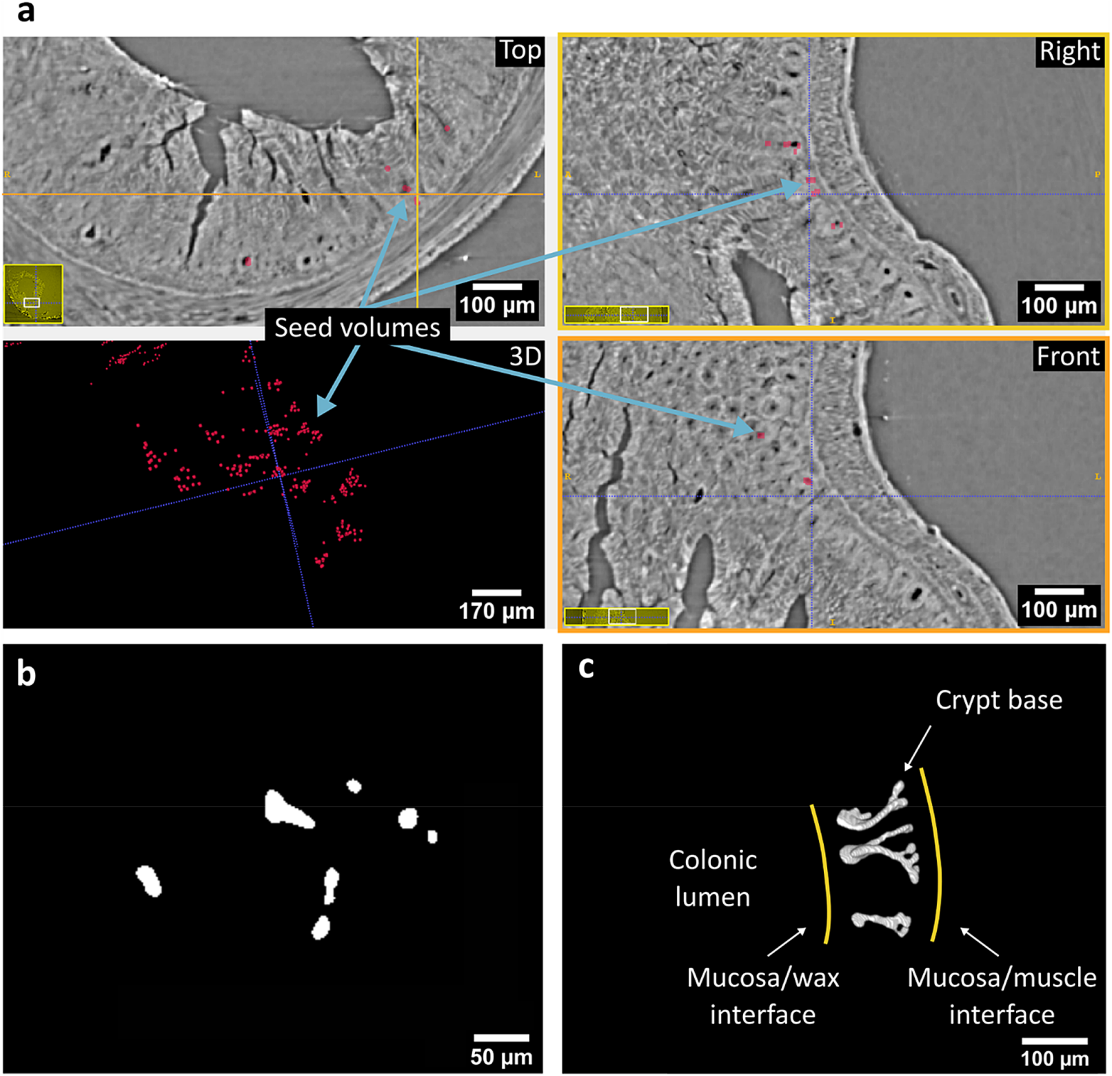
Budding crypt identification and 3D segmentation. (**a**) The viewport of ITK-SNAP is composed of four views (Top, Right, Front and 3D). The panels Top, Right and Front show orthogonal cross sections of the SR CT stack. The panels Right and Front show cross sections across the yellow and orange lines on the panel named Top, respectively. The panel named 3D shows the CT mask with the volumes of the back-mapped seed points (resulting from the inverse mapping between the 3D cyclorama and the CT data). The 3D seed volumes shown in the panel 3D, which are overlaid on the CT stack in the panels named Top, Right, and Front, are used to initialise the segmentation and expand it throughout the crypt lumens using region growing. The panels named Top, Right, and Front only show a zoomed-in view of the respective orthogonal cross sections. The white rectangles within the yellow panes on the bottom left corners of the Top, Right, and Front panels show which part of the cross sections is displayed in the panel. (**b**) Each slice of the image stack produced by the segmentation procedure is a binary image, with pixels corresponding to the crypt lumens having the value of one (white) and zero (black) elsewhere. (**c**) A 3D rendering of the binary image stack reveals the budding crypts and their position in space within the tissue.

Next, we used ITK-SNAP (v2.8.0-beta) for isolation of the crypts in 3D. ITK SNAP [266] (www.itksnap.org), an open-source image processing application to segment 3D structures in image stacks, provides semi-automatic segmentation using active contour methods. It requires a set of seed volumes that are expanded over the 3D image by applying a region growing algorithm, which maintains a curve or active contour. The contour evolves under the influence of a region completion force that tends to expand the curve, and a smoothing factor that tends to make the curve less rugged. On each iteration step, ITK-SNAP reads the image grey values around the curve and decides how the curve needs to evolve in order to segment the crypts. We created the seed volumes for ITK-SNAP as a binary mask with a zero value for the background, and cubes of 5 × 5 × 5 pixels^3^ (or 11 × 11 × 11 μm^3^) with a value equal to one, centred on each of the points (inside the crypts) on the cyclorama. We subsequently mapped this cyclorama mask back onto the CT stack using the inverse of the computed mapping between the CT stack and the 3D cyclorama, resulting in a (binary) CT mask. The bit-depth of the CT stacks was reduced from 16 to 8 bit with grey-value clipping thresholds at 21,000 a.u. and 37,000 a.u. (derived using the ‘Auto-threshold tool’ in Fiji, with the default algorithm: ‘isodata’^47^). The back-mapped seed volumes resulting from the inverse mapping appear as red cubes in the 3D view, and as red overlaid areas on the orthogonal views of Fig. 5a. This 3D binary CT mask had the same dimensions as the CT stack of the colonic segment (1844 × 1844 × 301 pixels^3^ or 4.1 × 4.1 × 0.7 mm^3^), and was provided to ITK-SNAP as an initial image segmentation. We then initialised the region growing algorithm with these seed volumes and ran it for 200 iteration steps with a low-pass filter threshold of 117 a.u., a region completion force equal to 1, and a smoothing factor of 0.2. Upon completion, the region growing algorithm provided a 3D segmented stack composed of slices such as that shown in Fig. 5b, a 3D volumetric reconstruction of which is shown in Fig. 5c.

### Validation results: murine colon unrolling

The process of identifying budding crypts in the unrolled murine colons is shown in Fig. 4. An example of the electric field lines and contours (only $C = 5$ depth levels are shown here for visual clarity) resulting after the application of virtual electrical charges on the colonic boundaries is shown in Fig. 4b. Figure 4c provides a photorealistic 3D rendering that illustrates the sample’s boundaries and the re-slicing surface at 50% relative depth level, yielding the probed CT greyscale values. Figure 4d-f show segments of the 2D cycloramas at incremental relative depths (80%, 82%, 84%), which, being locally perpendicular to the colonic crypts, depict the colonic crypts as round profiles. For the untreated sample, the healthy crypts do not split, and hence, their cross sections are consistent throughout all cyclorama slices (see Supplementary Video 1). The AOM/DSS-treated mouse, however, exhibits crypt budding, which is readily visible in the 3D cyclorama by identifying the tissue depth where a single crypt cross section splits to more than one (see Supplementary Video 2).

Note that not only the original shape of the crypts has been preserved in the cyclorama, but also their relative location and orientation in space. This is only possible because our method avoids physical cutting, thus tissue distortions typical for conventional (2D) thin section-based histology^48^ are not an issue. Moreover, each of the 2D cycloramas representing internal tissue layers at a specific tissue depth can be further analysed, for instance to count the number of crypts and their number density. In other words, our 3D cyclorama method advances time-consuming traditional (2D) thin-section histology via the use of re-slicing onion-like surfaces that follow the 3D shape of the colon, while obtaining non-planar slices across the entire tissue block. This internal re-slicing is impossible in standard (2D) thin-section histology due to the nature of the involved (planar) physical sectioning. This capability provided by our method is the key enabling feature for in situ study of crypt budding in 3D.

When it comes to computational models of crypt budding, they have been confined to the study of the healthy case of crypt fission. This is partly because validation of mathematical models of the crypt is particularly difficult, either due to the lack of experimental data or the difficulty to measure certain model parameters^49,50^. Notably, recent studies of crypt budding relate the spatial concentration of proteins (including the APC, β-catenin, E-cadherin and survivin) and cell proliferation and differentiation (including stem and mucus-secreting cells) with the crypt budding process^51–53^. By employing non-destructive CT imaging of intact FFPE colon samples, our method facilitates a correlative approach where concurrent structural information (via CT) and functional information (via serial histology sections) is now possible, whereby for the first time in situ, 3D, structural and functional characterisation of crypt budding during the early stages of CRC can be performed.

## Discussion

Today, application areas of μCT and SR CT are broad, including archaeology, biology, chemistry, environmental science, forensics, material science and physics^15,54^. To physically unroll samples is often impossible or destructive^26^. On this account, non-destructive 3D imaging, such as confocal laser scanning microscopy, magnetic resonance imaging or X-ray CT, and subsequent digital unrolling is an alternative method to study the sample’s 3D structure. A method for digital unrolling of deformed tubes would be beneficial for a broad spectrum of scientific and industrial applications.

In the field of archaeology, deciphering of ancient scrolls^26–29^ or studying the wrapping material of mummified cats^55^ are prime examples where digital unrolling is beneficial and has been used in the past. Inscriptions on metallic scrolls (lead, silver, gold or alloys), though, extend over a certain depth within the metal^56^. As a result, existing approaches to unroll these samples are only applicable to relatively thin samples with close-to-uniform thickness. Hence, such cases could benefit from a volumetric unrolling approach, such as our cyclorama method, to extract the full 3D nature of the inscribed literals. Similarly, in industry, procedures for non-destructive testing of turbine blades or aircraft wings, which are examined for fatigue life faults and internal microcracks during manufacture and/or maintenance^54,57,58^, could also be facilitated by our 3D cyclorama method. Unrolling or flattening manufactured parts with challenging geometries could speed up the examination for 3D crack propagation and manufacturing defect distribution via effective visualisation of the sample’s volume.

Our 3D cyclorama method can facilitate visualising the internal structure of deformed cylindrical tubes of non-uniform thickness, yet the projected volumes should be interpreted with care. The 3D cyclorama is deformed both along contours and in radial direction, thus the scale is not preserved, resulting in anisotropic and non-uniform pixel/voxel sizes. This uneven scale implies that distances should not be measured on cycloramas. Nevertheless, the sample topology is always preserved, i.e., neighbouring features in the original volume will remain neighbours in the projected space (i.e., cycloramas) and vice versa. This originates in the construction of the re-slicing surfaces and the mapping, whereby we create the re-slicing surfaces based on (virtual) electric field lines that never overlap. We further make use of a mapping approach that offers two benefits. First, contours are mapped independently of each other, allowing implementation of the algorithm in a multi-threaded fashion to improve computational performance. Secondly, it creates cycloramas identical in size and shape, readily solving the subsequent registration problem, i.e., the problem of aligning 2D cycloramas from different depths of the tube, so that features extending over several layers match up and are represented as continuous entities within the 3D cyclorama. This unavoidably introduces image deformations across the entire image, resulting in wavy but continuous cycloramas as shown in Fig. 3e.

It is worth to investigate the possibility of employing alternative mapping strategies not addressed yet. Notably, as our approach treats each contour individually for the benefit of multithreading, this confines our abilities to impose certain properties on the 2D cycloramas that result from the mapping, such as minimal image deformation, maximal smoothness, etc. Approaches within the context of manifold embedding could be used to impose such desired properties for the mapping of the 3D re-slicing surfaces. Such methods are studied in the field of differential geometry^59^, where manifolds (topological spaces that are locally Euclidean) can be embedded in lower-dimensional equivalents.

In our case, the dimensionality of the re-slicing surface needs to be reduced from 3D to 2D. This mapping or embedding can be done in different ways, including the isometric mapping^60^ for minimal image deformation, or the moving least squares mapping^61^ for maximal smoothness, depending on the desired properties of the target space. For instance, isometric mapping^60^ computes the embedding that optimally preserves geodesic distances between the points on the manifold. This algorithm would preserve the shapes of features on the re-slicing surface, effectively minimising image deformation of feature shapes on the mapped space. However, it would not ensure consistency among the shape of all 2D equivalents since it does not necessarily yield rectangular images. This means that each 3D re-slicing surface would be mapped onto a 2D equivalent with different dimensions and shape. Hence, a subsequent step of registration among the 2D equivalents would be necessary to match up features in sequential 2D surfaces to combine them into a 3D stack depicting the unrolled volume. Our approach solves this problem by imposing identical shape and size for 2D cycloramas that are stacked to produce a 3D cyclorama, at the expense of the image deformation types discussed in section ‘Mapping: Generation of 3D cycloramas’.

The moving least squares approach^61^ computes a mapping that yields a mapped surface with maximal smoothness. This method requires the user to provide a small set of control point pairs (a point on the source surface and its mapped position) and creates a smooth surface that respects the given set. This method would be suitable to create rectangular 2D surfaces of identical size and shape. It is an elegant and mathematically sound method that is worth investigating and integrating with our approach of reforming the deformed tube into onion-like re-slicing surfaces using notions from electrostatics: The requirement of a set of input control point pairs in the formulation of the moving least squares approach could be conveniently integrated with the 3D cyclorama method given our definition of re-slicing surfaces as a set of contours made up of sequences of points. Control point pairs could be created by matching up points $\vec{s}_{{i,h,c}}$ from the re-slicing surface (Fig. 3b) with points $\vec{p}_{{i,h,c}}$ on the 3D cyclorama grid (Fig. 3d). Selecting a small set of control point pairs would provide enough flexibility for image deformation in both directions *h* and *l* (see Fig. 3d-e) in addition to the radial image deformation (direction *c*) already introduced through the definition of the relative depth levels (see Fig. 3b-d). This flexibility of image deformation in all directions would result in a smooth 3D image deformation field.

Although our current implementation for 3D cycloramas was developed to unroll deformed tubes, the mathematical framework can, in principle, be applied to digital volume flattening/straightening of thick sheets and scrolls of non-uniform thickness as well. To achieve this, the two boundary contours would need to be open (non-convex), following the shape of the sheet/scroll. Adaptation of our method to unroll scrolls of non-uniform thickness would advance the solution of digital unrolling of ancient papyri and amulets with written or inscribed text^26–29^, from a 2D approach to a fully 3D volume unrolling, revealing information hidden in deeper layers of a scroll.

## Conclusion

We developed a method (3D cyclorama) to non-rigidly unroll deformed tubes of non-uniform thickness. Virtual electrical charges were applied on the tube’s boundaries, and by deriving the resulting (virtual) electric field lines, we could generate internal onion-like 3D surfaces that followed the shape of the deformed tube. This approach of using notions from electrostatics to restructure a volume as internal re-slicing surfaces is our main contribution to the problem of volume unrolling. We showcased the utility of our 3D cyclorama method (validation) through application of digital unrolling of intact colons for the study of crypt budding, and two case studies in Supplementary S.2 and S.3 (segmentation of microvilli in the human placenta and digital flattening of a 3D-printed adhesive film, respectively), which present how our 3D cyclorama method can be applied to volumes imaged by dissimilar 3D imaging techniques.

Applications of our method, however, go further beyond the case studies we presented here. It can be useful for the analysis of digital volumes created by any 3D imaging technique, such as clinical CT, confocal laser scanning microscopy, electron tomography, light sheet microscopy, magnetic resonance imaging, X-ray CT, etc., with applications spanning a large range of fields, from archaeology with unrolling of ancient scrolls and mummified cats to biomedical research, where abnormal crypts are studied to better understand CRC.

## Supplementary Information

Supplementary Information

Supplementary Figure S1

Supplementary Figure S2

Supplementary Figure S3

Supplementary Figure S4

Supplementary Figure S5

Supplementary Figure S6

Supplementary Video 1.

Supplementary Video 2.

Supplementary Video 3.

## Data Availability

The data and software supporting this work are openly available from the University of Southampton institutional research repository at https://doi.org/10.5258/SOTON/D1407. The 3D cyclorama software developed through this work has been adapted as a Fiji plugin. The latest version of the plugin is made available through the Fiji update sites. For more information please visit https://imagej.github.io/plugins/3d-cyclorama.
