# Supplementary material for: 3D cyclorama for digital unrolling and visualisation of deformed tubes
Source: Sci Rep. 2021 Jul 19;11:14672. doi: 10.1038/s41598-021-93184-x (PMC8289852; doi:10.1038/s41598-021-93184-x)
Supplement: Supplementary file 1 — Supplementary Information [file 41598_2021_93184_MOESM1_ESM.docx]

**3D cyclorama for digital unrolling and visualisation of deformed tubes**

**Authors:** Charalambos Rossides^1,2^, Sylvia L. F. Pender^2, 3^, Philipp Schneider*^1,4^

**Affiliations:**

^1^Bioengineering Science Research Group, Faculty of Engineering and Physical Sciences, University of Southampton, Southampton, UK

^2^School of Clinical and Experimental Sciences, Faculty of Medicine, University of Southampton, Southampton, UK

^3^Institute for Life Sciences, University of Southampton, Southampton, UK

^4^High-Performance Vision Systems, Center for Vision, Automation & Control, AIT Austrian Institute of Technology, Vienna, Austria

**Funding sources:**

This study was supported by the Engineering and Physical Sciences Research Council (EPSRC) Doctoral Training Partnership (DTP) (EP/N509747/1), the Institute for Life Sciences, University of Southampton, Soutahmpton, UK, and Nikon X-Tek Systems Ltd, Tring, UK.

**Emails:**

Charalambos Rossides, MSc c.rossides@soton.ac.uk

Sylvia Pender, PhD s.pender@soton.ac.uk

Philipp Schneider, PhD p.schneider@soton.ac.uk

***Corresponding author:**

Philipp Schneider

University of Southampton

Highfield Campus

Building 7 (Lanchester) / Room 4031 – Mailpoint M7

SO17 1BJ, Southampton, UK

Email: p.schneider@soton.ac.uk

**Number of words:**

Abstract = 196

Supplementary = 3996

**Number of supplementary figures:**

Colour = 5

Black & white = 1

**Number of supplementary videos:**

Colour = 0

Black & white = 3

**Disclosures:**

All authors state that they have no conflicts of interest.

# Abstract

Colonic crypts are tubular glands that multiply through a symmetric branching process called crypt fission. During the early stages of colorectal cancer, the normal fission process is disturbed, leading to asymmetrical branching or budding. The challenging shapes of the budding crypts make it difficult to prepare paraffin sections for conventional histology, resulting in colonic cross sections with crypts that are only partially visible. To study crypt budding *in situ* and in 3D, we employ X-ray micro-computed tomography to image intact colons, and a new method we developed (3D cyclorama) to digitally unroll them. Here, we present, verify and validate our ‘3D cyclorama’ method that digitally unrolls deformed tubes of non-uniform thickness. It employs principles from electrostatics to reform the tube into a series of onion-like surfaces, which are mapped onto planar panoramic views. This enables the study of features extending over several layers of the tube’s depth, demonstrated here by two case studies: (i) microvilli in the human placenta and (ii) 3D-printed adhesive films for drug delivery. Our 3D cyclorama method can provide novel insights into a wide spectrum of applications where digital unrolling or flattening is necessary, including long bones, teeth roots and ancient scrolls.

# Keywords

Colorectal cancer, Crypt branching, Computed tomography (CT), 3D cyclorama, Digital unrolling, Digital flattening, Digital straightening.

# Supplementary

## S.1 Verification of the 3D cyclorama method

To verify the presented digital volume unrolling method, we generated a 3D digital phantom (see supplementary Figures S1-S4) in two steps. We first created a 2D grid template *in silico*, which was used to create both a deformed cylindrical tube (digital phantom) and a 3D template stack (ground truth). The digital phantom (see supplementary Figure S2 and Figure S4) was created by rolling and stretching the 2D grid template (see supplementary Figure S1) by means of an analytical mapping we devised (see supplementary Figure S3). The 3D template stack or ground truth (see supplementary Figure S4) was built as a planar version of the digital phantom using the same 2D grid template. We then applied our 3D cyclorama method to unroll the phantom volume and compare the resulting 3D cyclorama (see supplementary Figure S4) visually and quantitatively with the 3D template stack as ground truth. Next we describe this process in detail.


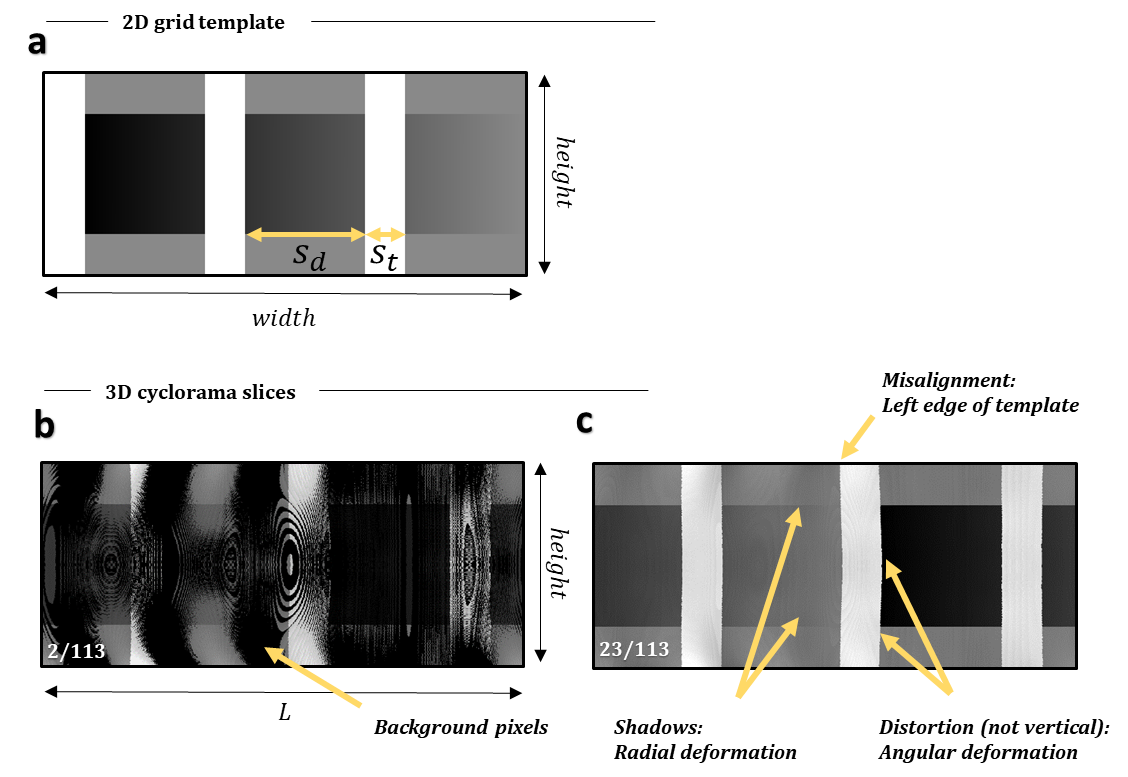


**Figure S1: 2D grid template and slices of the 3D cyclorama after unrolling of the digital phantom**

(a) The 2D grid template used to create the digital phantom is composed as a greyscale gradient with superimposed horizontal and vertical bands. These serve both as visual cues and as features for quantitative evaluation of the image deformation after unrolling. (b-c) Two slices of the 3D cyclorama after unrolling the digital phantom at 3% (2/113) and 21% (23/113) relative depth level. Dark patches on the 3% (2/113) slice emerge due to probing of background pixels during the mapping of superficial surfaces close to the inner boundary. Angular image deformation appears as curved vertical lines on the 21% (23/113) slice, while image deformation in the radial direction appears as shadows due to probing of pixels from neighbouring depths. The cycloramas were aligned with respect to the 2D template, since the cutting edge is defined azimuthally by the arbitrary first point of the contours.

First, we created a 2D grid template image $g_{h,j}$ with pixel greyscale values according to the following rule:

| $g_{h,j}=offset+j\cdot height+h$, $j=0\ldots width-1$, $h=0\ldots height-1$ , | (S1) |
| --- | --- |

where $offset>0$ is an arbitrary positive integer and $width$ and $height$ are the image width and height in number of pixels, respectively. In a next step, two single-valued horizontal and three single-valued equidistant vertical stripes with thickness $s_{t}$ and distance $s_{d}$ were superimposed in order to create a rectangular grid (see supplementary Figure S1). The arbitrary offset was chosen to be positive so that after rolling of the template onto a deformed cylindrical tube (see supplementary Figure S2), it can easily be differentiated from the background (greyscale value of background = 0). The two indices $j$ and $h$ introduce a gradient (see supplementary Figure S1) to create features of varying 16-bit greyscale values, necessary for quantitative comparison between the 3D cyclorama (unrolled version of the deformed cylindrical tube) and the 3D template (ground truth). The horizontal and vertical boundaries of the stripes create visual cues that help assess the amount of image deformation in the cycloramas in a qualitative manner. We implemented the 2D grid template image $g_{h,j}$ (see supplementary Figure S1) using the following sizes in number of pixels: $offset=296,$ $width=L=1200,$ $height=H=500$, $s_{d}=300$, $s_{t}=100$.

**
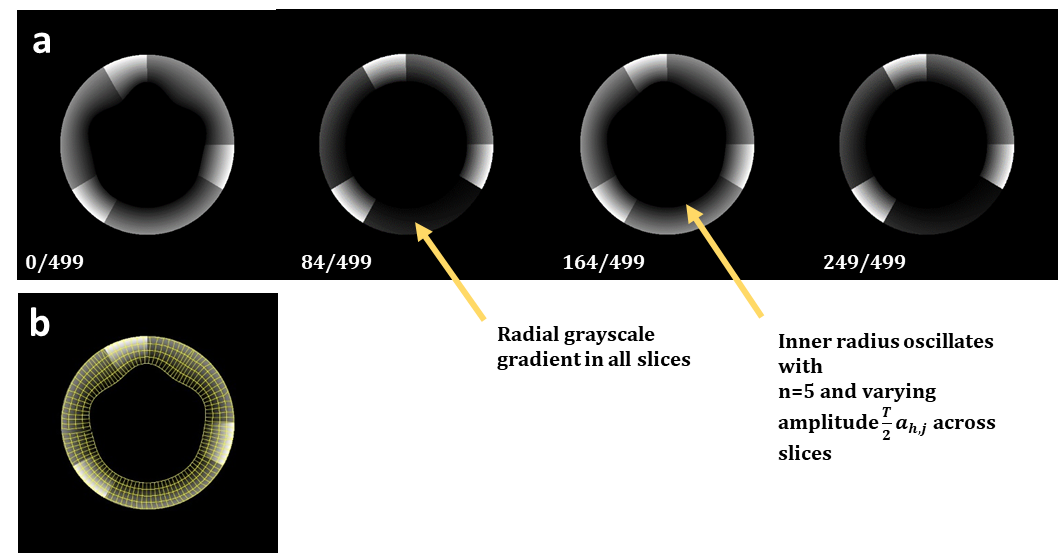
**

**Figure S2: Cross sections of the 3D digital phantom**

(a) Cross sections of the digital 3D phantom at incremental heights ($0\leq h\leq499$). The inner radius $r_{h,j}$ is modulated by a sinusoid of changing amplitude and frequency, creating a phantom shape that is challenging to unroll. Greyscale values of the phantom also change across its wall thickness, with the outer side being brighter (larger grey values towards the outer boundary) than the inner side (smaller grey values towards the inner boundary). (b) Electric field lines and contours used to unroll the digital 3D phantom (only $C=5$ depth levels are shown here for visual clarity).

We defined the geometry of a deformed tube (digital phantom) by the outer boundary that was described as a circle with constant radius across all heights of the tube (see supplementary Figure S3):

| $x_{out,j}=x_{c}+ \left\lfloor R\cdot cos(\theta_{1,j}) \right\rfloor$ , | (S2) |
| --- | --- |
| $y_{out,j}=y_{c}+ \left\lfloor R\cdot sin(\theta_{1,j}) \right\rfloor$ ,$j=1\ldots\left\lfloor2\pi R \right\rfloor$ | (S3) |

with outer radius $R$, centre of the circle${(x}_{c}, y_{c})$, azimuthal angle $\theta_{1,j}=[0, 2\pi)$ and $\left\lfloor\right\rfloor$ denoting the floor function, while the inner boundary at a certain height $h$ was defined as follows:

| $x_{in,h,j}=x_{c}+ \left\lfloor r_{h,j}\cdot cos(\theta_{1,j}) \right\rfloor$ , | (S4) |
| --- | --- |
| $y_{in,h,j}=y_{c}+ \left\lfloor r_{h,j}\cdot sin(\theta_{1,j}) \right\rfloor$ , | (S5) |
| $r_{h,j}=R-\frac{T}{2}a_{h,j}$ , | (S6) |
| $a_{h,j}=\left( \frac{1+sin(n\cdot\theta_{1,j}+sin(\varphi_{1,h}))}{2}\cdot\frac{1+\sin\left( \varphi_{2,h} \right)}{2}\cdot\frac{1+sin(\theta_{2,j})}{2} \right)+1, j=1\ldots width, h=1\ldots height$ , $n\mathbb{\in N}$ , | (S7) |
| $r_{h}:=\min_{j} r_{h,j}=R-T\leq r_{h,j}\leq\max_{j} r_{h,j}=R-\frac{T}{2}$ , $j=1\ldots width$ , $h=1\ldots height$ , | (S8) |

with the real maximal tube thickness $0<T<R$ and $width=\left\lfloor2\pi R \right\rfloor$. The real amplitude $a_{h,j}\in[1,2]$ modulates the inner radius $r_{h,j}$ of the tube ($r_{h}$: minimal inner radius at height $h$). For $a_{h,j}$, changes with angles $\theta_{1,j}=[0, 2\pi)$ and $\theta_{2,j}=[-\pi, \pi)$ are both linear azimuthal angle sequences with number of elements equal to $width$, while $\varphi_{1,h}=[0, \pi)$ and $\varphi_{2,h}=[-\pi, 0)$ are polar angle sequences with number of elements equal to $height$. The inner radius $r_{h}$ takes values in the range of $\left[ R-T, R-\frac{T}{2} \right]$, yielding a varying tube thickness of ${R-r}_{h,j}=\frac{T}{2}a_{h,j}\in$ $\left[ \frac{T}{2}, T \right]$, as if the tube was radially ‘stretched’ and ‘compressed’, leading to an azimuthally changing tube thickness. Finally, the integer $n$, which is multiplied with $\theta_{1,j}$, defines the frequency with which the inner radius oscillates (see supplementary Figure S2). In essence, the modulating amplitude $a_{h,j}$ enforces the tube’s or phantom’s thickness to change locally, creating a shape that is challenging to unroll. We arbitrarily selected $n=5$ to create the phantom shown in supplementary Figure S2. A 3D image stack $G_{h,\rho_{h,j}, \theta_{h,j}}$, which represents a deformed tube with the geometry described above, was then created by probing the grey values of the 2D grid template $g_{h,j}$ as follows:

| $G_{h,\rho_{h,j}, \theta_{h,j}}=w_{\rho_{h,j}}\cdot g_{h,j}, j=1\ldots width, h=1\ldots height, w_{\rho_{h,j}}\mathbb{\in R}$ , | (S9) |
| --- | --- |

where $w_{\rho_{h,j}}=\left[ 1, R-r_{h,j} \right]=\left[ 1, \frac{T}{2}a_{h,j} \right], \frac{T}{2}a_{h,j} \in\left[ \frac{T}{2}, T \right]$ is a radius-dependent weight that introduces a linear scaling of the grey values of the 2D grid template across the depth of the tube (see supplementary Figure S2), which is proportional to the varying tube thickness (see supplementary Figure S3). The radius $\rho_{h,j}=\left[ r_{h,j},R \right]=\left[ R-\frac{T}{2}a_{h,j},R \right]$ ranges from the inner to the outer boundaries of the deformed tube $G_{h,\rho_{h,j}, \theta_{h,j}}$ at height $h=[1, height]$ with the azimuthal angle $\theta_{h,j}$ defined as follows:

| $\theta_{h,j}=\theta_{1,j}+\max\left\{ {R-\frac{T}{2}-\rho}_{h,j}, 0 \right\}\cdot\left( a_{h,j}-\frac{3}{2} \right)$ . | (S10) |
| --- | --- |

Equation (S9) defines a mapping between index $j$ on the grid template and azimuthal angle $\theta_{h,j}$ on the digital phantom, introducing a non-rigid image deformation. Radial and angular image deformations are introduced to the deformed tube by Equations (S6) and (S10), respectively. These image deformations mimic the case where a perfect tube with outer radius $R$ and inner radius $R-\frac{T}{2}$ is non-rigidly deformed by stretching it inwards at specific azimuthal angles. The non-rigid image deformation that is introduced in this case is implemented analytically by offsetting the angle $\theta_{1,j}$ by $\max\left\{ {R-\frac{T}{2}-\rho}_{h,j}, 0 \right\}\cdot\left( a_{h,j}-\frac{3}{2} \right)$. The first factor $\max\left\{ {R-\frac{T}{2}-\rho}_{h,j}, 0 \right\}$ makes this offset non-zero when the tube’s thickness ${R-r}_{h,j}=\frac{T}{2}a_{h,j}\in$ $\left[ \frac{T}{2}, T \right]$is larger than $\frac{T}{2}$ and zero elsewhere, thus introducing image deformations only at the inner half of the tube’s thickness (see supplementary Figure S3). The second factor $\left( a_{h,j}-\frac{3}{2} \right)$ of this offset shifts the mean value of $a_{h,j}$ to zero, thus ensuring that the template is compressed ($\theta_{h,j}$ follows $\theta_{1,j}$) when the offset is negative and on the other hand, stretched ($\theta_{h,j}$ precedes $\theta_{1,j}$) when the offset is positive (see supplementary Figure S3). Since the deviation of the inner radius $r_{h,j}=R-\frac{T}{2}a_{h,j}$ from the midline at radius$R-\frac{T}{2}$ is proportional to the offset $\max\left\{ {R-\frac{T}{2}-\rho}_{h,j}, 0 \right\}\cdot\left( a_{h,j}-\frac{3}{2} \right)$, the degree of compression and stretching follows a similar pattern. We implemented the digital 3D phantom with $R=190$ pixels, $T=114$ pixels, $n=5$, $x_{c}=x_{y}=287$ pixels, resulting in an image stack of 500 slices, with a dimension of 573×573 pixels^2^ each.


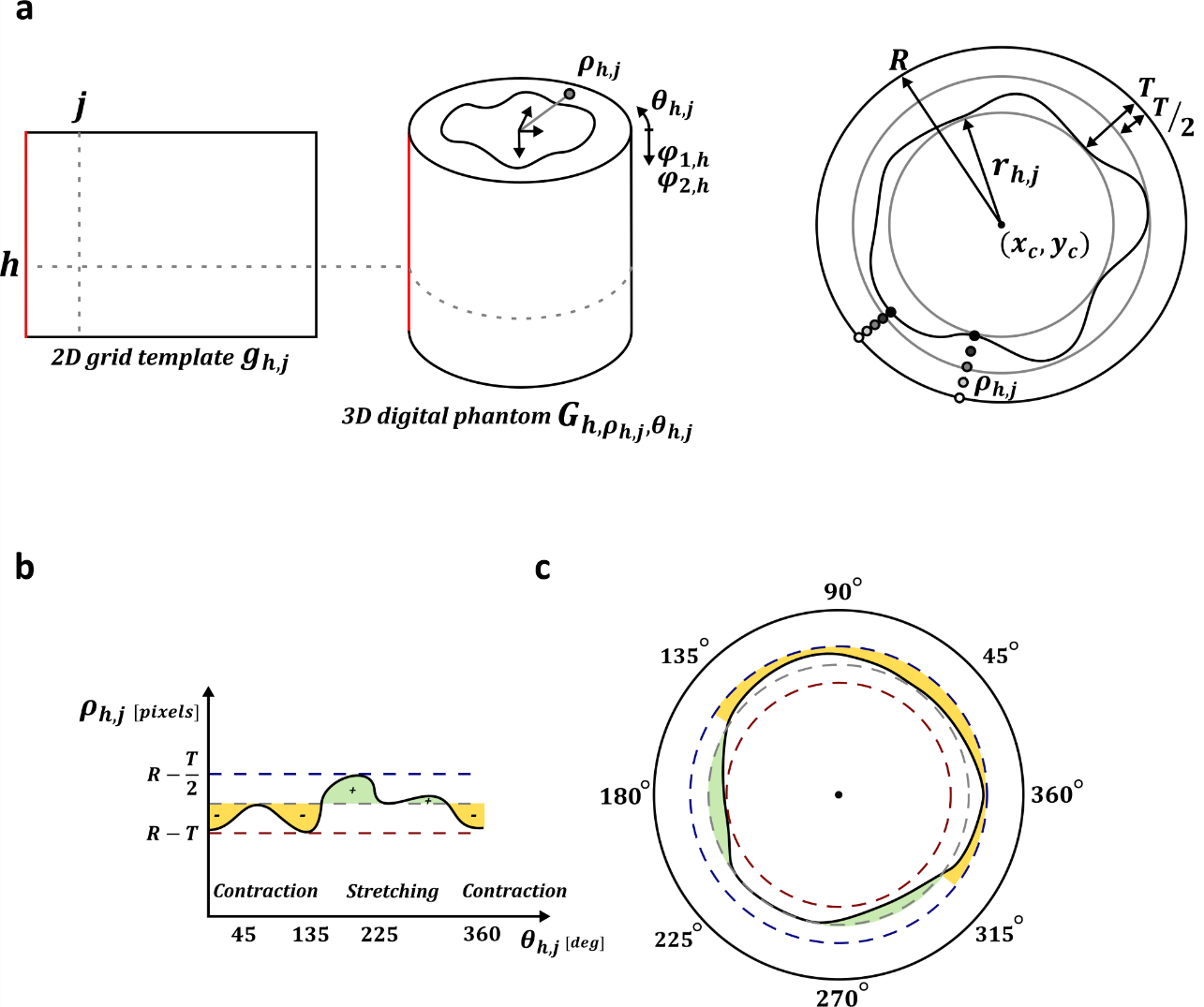


**Figure S3: Mathematical description of the 3D digital phantom**

(a) A 3D digital phantom was created as deformed tube (3D image stack $G_{h,\rho_{h,j}, \theta_{h,j}}$) by rolling a 2D (rectangular) grid template image $g_{h,j}$ and mapping points $\left( h,j \right)$ to points $\left( h,\rho_{h,j}, \theta_{h,j} \right)$. (b) The wall of the digital phantom has a fixed outer radius $R$ and an inner radius $r_{h,j}$ that follows a sinusoid of varying angular frequency and amplitude (minimal inner radius $r_{h}:=\min_{j} r_{h,j}=R-T\leq r_{h,j}\leq\max_{j} r_{h,j}=R-\frac{T}{2}$), giving rise to a minimum and maximum thickness $\frac{T}{2}$ and $T$ of the deformed tube, respectively. The tube is assigned by linearly scaled greyscale values, where brightness levels are proportional to the radius $\rho_{h,j}$ at points $\left( h,\rho_{h,j}, \theta_{h,j} \right)$ through sampling of the grid template $g_{h,j}$. (c) The undulating inner radius $r_{h,j}\in\left[ R-T, R-\frac{T}{2} \right]$ results in a varying tube thickness ${R-r}_{h,j}=\frac{T}{2}a_{h,j}\in$ $\left[ \frac{T}{2}, T \right]$. The grey midline of this band at radius $\frac{1}{2}\left( R-\frac{T}{2}+r_{h} \right)=r_{h}+\frac{T}{4}$ can be considered as the boundary of a tube that has been stretched and contracted by pulling and pushing its inner boundary, resulting in contracted (‘$-$’ : yellow) and stretched (‘$+$’: green) volumes inside the wall of the digital phantom. This figure was created with Inkscape ^1^.

Finally, we created the 3D template stack (ground truth) using the same 2D grid template$g_{h,j}$:

| $g_{h,j,k}=w_{k}\cdot g_{h,j}, k, w_{k}\mathbb{\in N}$, | (S11) |
| --- | --- |

where $h=[1, height]$, $j=[1, width$], $k=[1, T]$ and $w_{k}=k$ is a depth-dependent weight that introduces a linear scaling of the grey values of the grid template across the depth $k$ of the 3D template stack. The implementation of this 3D template stack had 114 slices with a dimension of 1200×500 pixels^2^ each.

We used this 3D template stack $g_{h,j,k}$(see supplementary Figure S1 and Figure S4) as the ground truth to evaluate the result of our 3D cyclorama method when applied to unroll the digital 3D phantom $G_{h,\rho_{h,j},\theta_{h,j}}$. We quantified the quality of unrolling by estimating the non-rigid transform that maps the resulting 3D cyclorama onto the 3D template stack, by calculating and visualising the local volume change it introduced. To this end, we used *Elastix* ^2^, an open source collection of image registration algorithms, to non-rigidly register the two 3D image stacks. The software estimates the optimal transformation that maps the 3D cyclorama on the 3D template (see supplementary Figure S4) to maximise their mutual information as an image matching metric. Elastix provides the Jacobian determinant $\alpha$ of the transformation at each point in space, which quantifies the volume of a transformed unit cube. In other words, the Jacobian determinant shows how much the space has been stretched or compressed at each point of the transformed 3D cyclorama to fit the 3D template, imposed by our digital unrolling at each point in 3D. Identity of the Jacobian determinant means an exact match, i.e., the 3D cyclorama exactly matches the phantom, while a Jacobian determinant value $\alpha\neq1$ means that the volume of the 3D cyclorama at that voxel is $\alpha$ times smaller ($\alpha<1$) or larger ($\alpha>1$) than the phantom. We chose to use the Jacobian determinant (rather than measuring the absolute difference between the 3D cyclorama and the 3D phantom) to make the quantification independent of the absolute greyscale values.

We defined the boundary contours by absolute thresholding of the digital phantom’s grey values shown in supplementary Figure S2 and Figure S4. Unrolling was performed with $C=114$ depth levels (supplementary Figure S2 shows $C=5$ depth levels for visual clarity), $I=100$ point-long contours, an electric field search window of 31 points ($w=15$), electric field line segment size $\delta=2$ pixels, maximum number of steps $K_{max}=57$, minimum electric field line variance $V_{min}=10$, rigidity equal to 1, interpolation interval $m=50$, and rotation axis origin $O=(300, 300)$. The resulting 3D cyclorama’s length was $L=1191$pixels, the height was $H=500$pixels, and the depth was $C=114$slices. Angular image deformations (see supplementary Figure S1) appear as geometrical distortion along the horizontal axis of the cycloramas. Radial image deformations appear as shadows in cycloramas as greyscale values from neighbouring depth levels $c$ of the 3D phantom, resulting in a diffuse grey level distribution observed in the 2D cycloramas (see supplementary Figure S1).

A few image pre-processing steps needed to be performed prior to comparing the 3D cyclorama quantitatively and visually with the 3D template stack as ground truth, for verification of the 3D cyclorama method. As contours begin azimuthally at an arbitrary first point, the 3D cyclorama is not necessarily aligned with the 3D template (see supplementary Figure S1). Thus, we firstly manually aligned the two stacks by rolling the 3D cyclorama (cropping the left part and attaching it to the right) so that the left side of the 3D cyclorama was aligned with the left side of the 3D template. Cycloramas close to the deformed tube’s boundaries partially included pixels of the background that appeared as distinct dark patterns (see supplementary Figure S1). We thus removed cyclorama slices (first four and last two depth levels $c=0,1,2,3$ and $c=112,113$) that have previously contained background (black) pixels from both the 3D cyclorama and the 3D template. Finally, as the cyclorama’s length $L$ is arbitrarily determined based on the length of the longest contour $cont_{h,c}$, it was slightly shorter than the 3D template (1191 instead of 1200 pixels). Therefore, we scaled the 3D cyclorama (*scal*e function in Fiji with bicubic interpolation) to fit the template dimensions, in order to obtain a better initialisation of the subsequent registration process and ensure convergence of the iterative registration algorithm in Elastix.


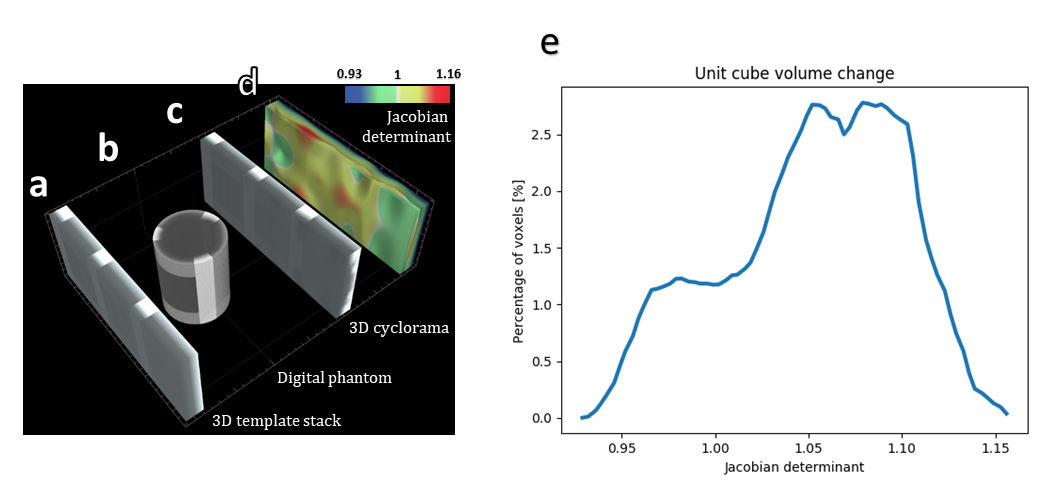


**Figure S4: Verification of the cyclorama method using a digital 3D phantom**

(a) The 3D template stack (ground truth) or digital 3D phantom consists of a volume with greyscale gradients making each voxel (except those on the vertical bands) distinct. (b) The digital phantom was created by digitally rolling a 2D grid template onto a deformed tube that is defined analytically. (c) The 3D cyclorama after unrolling, manual alignment (and scaling) to the 3D phantom. (d) The Jacobian determinant of the mapping shows the degree of volume change at each point in space. Identity of the Jacobian determinant means that the 3D cyclorama exactly matches the phantom, while a Jacobian determinant value $\alpha\neq1$ means that the volume of the 3D cyclorama at that voxel is $\alpha$ times smaller ($<1$) or larger ($>1$) than the phantom. (e) Non-rigid registration of the 3D cyclorama onto the 3D phantom returned the Jacobian determinant at each point of the 3D template. The mean value $\alpha=1.05$ of the Jacobian determinant distribution showed a general preference to preserve the volume. However, a skew to the right showed a trend of volume enlargement (up to 1.16 times). In this case, the maximum contraction was given by a Jacobian determinant of $\alpha=0.93$. (a-d) were created using VGStudio Max ^3^.

The registration process returned a 3D image stack with each voxel representing the Jacobian determinant at the corresponding point of the 3D template. A visual representation of the image deformation pattern (the value $\alpha$ of the Jacobian determinant) is provided in supplementary Figure S4, which shows that the image deformation was larger on the side of the inner boundary of the tube. The mean value $\alpha=1.05$ of the Jacobian determinant distribution (see supplementary Figure S4) shows a general preference to preserve the volume. However, a skew to the right shows a trend of volume expansion (up to 1.16 times), while the maximum contraction was given by a Jacobian determinant of $\alpha=0.93$. The trend for volume expansion (Jacobian determinant $\alpha>1$) shows that our method produces cyclorama stacks where the volume is locally enlarged. The root of this observation is our choice of mapping each contour onto a template whose length is equal to the greatest length among all contours. As discussed in section ‘2.3 Mapping: Generation of 3D cycloramas’, the mapping from a deformed tube onto an unrolled volume is not unique. Therefore, certain choices made to design the mapping method (e.g., preserving geodesic distances, mapping onto the longest contour, etc.) affect the characteristics of the unrolled volume. In our case, there is a slight preference for volume expansion. Volume contraction ($\alpha<1$) is introduced due to the radial image deformation (see supplementary Figure S3) imposed by our reformation of the volume into a series of re-slicing surfaces. The number of depth levels $C$ essentially defines whether the volume will be expanded or contracted in the radial direction. This happens because one 2D cyclorama with a thickness of one pixel is created for each depth level, resulting in a 3D cyclorama of depth equal to $C$. Therefore, when the number of depth levels $C$ is smaller than the maximum local thickness $T$, the volume of the deformed tube will be expanded radially so that the 3D cyclorama depth matches the maximum local thickness $T$. Similarly, the deformed tube volume will be contracted radially when $C$ is larger than $T$. In our case, where $C=T=114$, the observed contraction originates in rounding errors, where the same voxel of the digital phantom is sampled by two neighbouring contours. The fact that volume expansion/contraction depends on the choice of an input parameter (number of depth levels or contours $C$) means that the image deformation pattern would change accordingly and thus, as discussed in section ‘4. Discussion’, any quantitative measures derived from cycloramas must be interpreted with care.

## S.2 Case study: Human placenta

As our cyclorama method is agnostic of the origin of the specific 3D image dataset under investigation, it can be used to unroll image stacks that were created using any 3D imaging technique. Palaiologou and colleagues ^4^ employed serial block-face scanning electron microscopy (SBF SEM) to study the role of the human placenta’s microvilli in 3D. The placenta is the organ that connects the mother and her foetus, and it is responsible for the nutrient exchange and waste disposal between maternal and foetal blood circulation. Terminal villi on the microvillous membrane of the human term placenta are thought to be the major site of nutrient exchange through direct contact with the maternal blood ^4^. Technical challenges during 3D quantification include the fact that microvilli are highly convoluted, as well as the fact that the sample can be tilted arbitrarily during embedding (i.e., not aligned with the axis perpendicular to the image plane). On this account, segmentation of microvilli from SBF SEM data sets is challenging. We show here how our 3D cyclorama method can be used to work around this problem.

Term placental tissue was collected with written informed consent and ethical approval from the Southampton and Southwest Hampshire Local Ethics Committee (11/SC/0529), and a villous sample was imaged in 3D using SBF SEM ^4^ with a cutting thickness of 50 nm ($z$-direction) and nominal in-plane resolution of 4.2×4.2 nm^2^ ($xy$ plane) (volume of 2806×2684×134 pixels^3^ or 11.8×11.3×6.7 μm^3^) (see supplementary Figure S5).

The 3D image stack was scaled by ($x\cdot0.20, y\cdot0.20, z\cdot2.38$) using the *scale* function in Fiji ^5^ (ImageJ version 2.0.0-rc-68/1.52g, Java 1.8.0_66) with bicubic interpolation in order to mimic an isotropic voxel size (of 21 nm) and reduce the size of the stack to 561×536×319 pixels^3^ (with an unchanged volume of 11.8×11.3×6.7 μm^3^) shown in supplementary Figure S5. Since the microvillous membrane is not a tubular structure, we defined the boundary contours such that the membrane constituted a part of a deformed tube. A subset of 221 slices with dimensions of 561×536 pixels^2^ from the SBF SEM stack ($xy$ plane) was unrolled (see supplementary Figure S5) with $C=100$ depth levels, $I=50$ point-long contours, an electric field search window of 31 points ($w=15$), electric field line segment size $\delta=2$ pixels, maximum number of steps $K_{max}=75$, minimum electric field line variance $V_{min}=50$, rigidity equal to 1, interpolation interval $m=20$ and rotation axis origin $O=(450,450)$. The resulting 3D cyclorama’s length was $L=1698$pixels, the height $H=221$pixels, and the depth $C=100$slices.


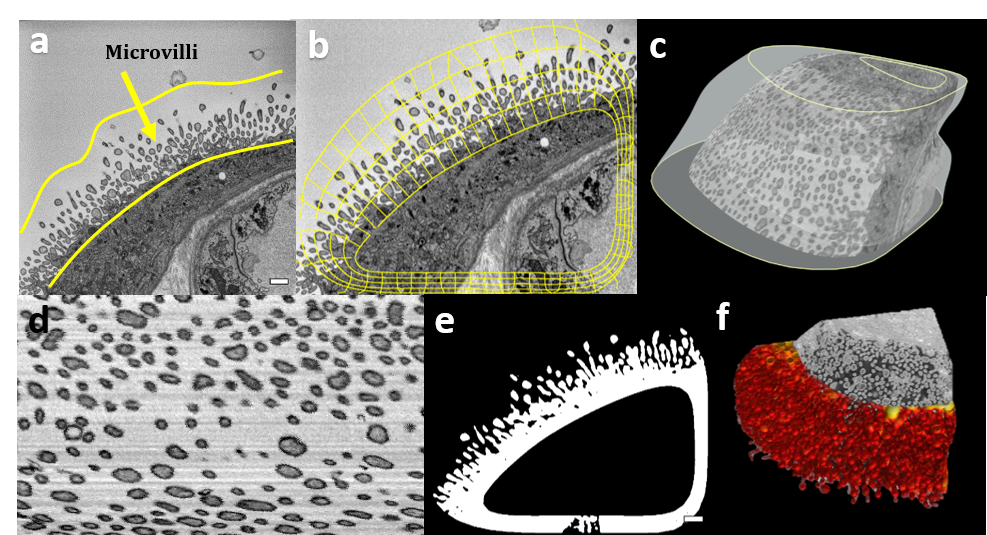


**Figure S5: 3D cyclorama to segment placental microvilli**

(a) A section from a SBF SEM stack of a human placenta. (b) Virtual electric field lines and the resulting contours were defined such that the membrane (enclosed in the two yellow lines in a) constituted part of a deformed cylindrical tube. (c) SBF SEM grey values are shown at 50% relative depth level, while boundary surfaces are shown transparent. (d) Placental microvilli on one 2D cyclorama. (e) 3D segmentation of the placental microvilli. (f) A photorealistic 3D rendering of the segmented microvilli (red) and the SBF SEM stack (grey). (a & e) Scale bars are equal to 1 μm. (c) was created using Mayavi ^6^. (f) was created using VGStudio Max ^3^.

The contours shown in supplementary Figure S5 (only $C=5$ depth levels are shown for visual clarity) were used to create re-slicing surfaces perpendicular to the microvilli. Supplementary Figure S5 provides a photorealistic 3D rendering that illustrates the sample’s boundaries and the re-slicing surface at 50% relative depth level, yielding the probed SBF SEM greyscale values, where the tilt of these surfaces reveals the shape of the sample. After unrolling of the microvilli, their cross sections become visible on the cyclorama image (see supplementary Figure S5), while the experimental tilt of the embedded sample does not influence the shape of these cross sections. The microvilli were then segmented on the 3D cyclorama and mapped back onto the initial volume (see supplementary Figure S5). This allowed visualising the sample and its complex shape in 3D (see supplementary Figure S5).

Our cyclorama method could play an important role in the segmentation process of microvilli, which is markedly difficult due to the peculiarities of the microvillous membrane’s shape in 3D and the alignment of the embedded sample for imaging. This case study shows that 3D cycloramas can be computed on 3D data, which has been retrieved from any 3D imaging technique.

## S.3 Case study: Pharmaceutical film

Applications of digital volume unrolling are not confined to biological samples. This case study shows how our 3D cyclorama method could be useful in the morphological assessment of 3D-printed adhesive films for drug delivery applications. Gioumouxouzis and colleagues ^7^ and Eleftheriadis and colleagues ^8^ employed fused deposition modelling (FDM 3D printing) in order to develop personalised drugs with unique properties and drug release behaviour. Eleftheriadis and colleagues subsequently evaluated the 3D-printed pharmaceuticals using μCT to compare their structure, when compared to the initial 3D design ^8^. This process becomes challenging when the 3D-printed object is flexible, as it is the case for certain pharmaceutical films that the authors are developing for time-controlled drug administration ^8^. The 20×20×0.8 mm^3^ plain film shown in supplementary Figure S6 is composed of poly(vinyl alcohol) as core polymer and plasticiser, and is designed for unidirectional drug release. μCT imaging resulted in a stack of 1000×161×901 pixels^3^ at an (isotropic) pixel size of 22 μm (volume of 22.0×3.5×19.8 mm^3^). Since the pharmaceutical film was curved (see supplementary Figure S6), it is difficult to quantify the deposition quality of the different drug layers. To this end, we applied our 3D cyclorama method to digitally flatten the film. As the pharmaceutical film was not tubular, we generated the boundary contours around it, such that the film became a section of a deformed tube, similarly to the previous case study (section ‘S.2 Case study: Human placenta’).

The μCT stack of 901 slices with dimensions of 1000×161 pixels^2^ was unrolled (see supplementary Figure S6) with $C=50$ depth levels, $I=100$ point-long contours, an electric field search window of 31 points ($w=15$), electric field line segment size $\delta=2$, maximum number of steps $K_{max}=50$, minimum electric field line variance $V_{min}=10$, rigidity equal to 1, interpolation interval $m=50$, and rotation axis origin $O=(340, 40)$. The resulting 3D cyclorama’s length was $L=2025$pixels, the height $H=901$pixels, and the depth $C=50$slices.


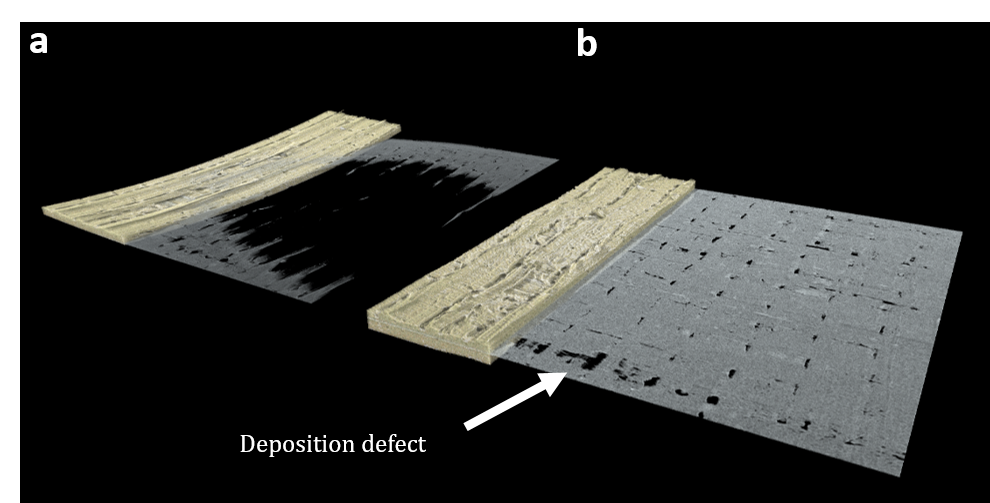


**Figure S6:** **3D cyclorama to unroll a pharmaceutical film**

(a) A curved 3D-printed adhesive film for drug delivery imaged by μCT, resulting in planar slices (grey) that only partially illustrate the film. (b) Flattening onto a 3D cyclorama can undo the curvature and allow structural analysis of the pharmaceutical film layers, revealing deposition defects such as gaps in drug distribution (arrow). (a&b) were created using VGStudio Max ^3^.

The 3D cyclorama (see Supplementary video 3) enables the quantification of the quality of drug deposition in each layer of the pharmaceutical film, as shown in Figure S6. In other words, after digital flattening, the 3D cyclorama stack enables examination of individual layers at incremental depths of the pharmaceutical film. Supplementary Figure S6 shows a deposition defect (gap in drug distribution) on a 2D cyclorama emerging out of a photorealistic rendering of the corresponding 3D cyclorama. This case study shows how the 3D cyclorama method can be employed to flatten 3D image stacks, not necessarily portraying biological samples, and to qualitatively characterise structures at different layers of the sample. Naturally, the inverse mapping could be utilised for quantitative morphometric analysis.

# Supplementary video legends

**Video 1: 3D cyclorama of untreated murine colon**

X-ray micro-computed tomographic imaging of murine colons and subsequent digital unrolling allows studying the 3D morphology of the colon in its original 3D shape. Sequential slices of the 3D cyclorama reveal tissue layers at incremental depths of the colon. Colonic crypt cross sections appear as dark circular spots, which can be traced through the tissue layers to study the crypt shape in 3D.

**Video 2: 3D cyclorama of AOM/DSS-treated murine colon**

Sequential slices of the 3D cyclorama reveal tissue layers at incremental depths of the colon. Colonic crypt cross sections appear as dark spots, which can be traced through the tissue layers to study the crypt shape in 3D. Crypt budding is readily identified where a single cross section splits to more than one cross sections in sequential slices.

**Video 3: 3D cyclorama of pharmaceutical film**

A 3D-printed adhesive film for personalised drug delivery, manufactured with fused deposition modelling (FDM 3D printing) was digitally flattened with the 3D cyclorama method. After digital flattening, the 3D cyclorama stack enables examination of individual layers at incremental depths of the pharmaceutical film. The X-ray micro-computed tomography image data was kindly provided by Dimitrios G. Fatouros, Georgios K. Eleftheriadis, and Orestis L. Katsamenis.

# References

1 Inkscape v. 0.92.4. https://inkscape.org.

2 Klein, S., Staring, M., Murphy, K., Viergever, M. A. & Pluim, J. P. Elastix: a toolbox for intensity-based medical image registration. *Ieee T Med Imaging* **29**, 196-205 (2010).

3 VGStudio Max, v. 2.1. https://www.volumegraphics.com/en/products/vgstudio-max.html.

4 Palaiologou, E. *et al.* Serial block-face scanning electron microscopy of erythrocytes protruding through the human placental syncytiotrophoblast. *Journal of Anatomy* **231**, 634-637, doi:10.1111/joa.12658 (2017).

5 Schindelin, J. *et al.* Fiji: an open-source platform for biological-image analysis. *Nature Methods* **9**, 676, doi:10.1038/nmeth.2019 (2012).

6 Ramachandran, P. & Varoquaux, G. Mayavi: 3D visualization of scientific data. *Computing in Science & Engineering* **13**, 40-51 (2011).

7 Gioumouxouzis, C. I. *et al.* Controlled Release of 5-Fluorouracil from Alginate Beads Encapsulated in 3D Printed pH-Responsive Solid Dosage Forms. *Aaps Pharmscitech* **19**, 3362-3375, doi:10.1208/s12249-018-1084-2 (2018).

8 Eleftheriadis, G. K. *et al.* Unidirectional drug release from 3D printed mucoadhesive buccal films using FDM technology: In vitro and ex vivo evaluation. *Eur J Pharm Biopharm* **144**, 180-192, doi:10.1016/j.ejpb.2019.09.018 (2019).
