# Supplementary figures and images for: 3D cyclorama for digital unrolling and visualisation of deformed tubes
Source: Sci Rep. 2021 Jul 19;11:14672. doi: 10.1038/s41598-021-93184-x (PMC8289852; doi:10.1038/s41598-021-93184-x)

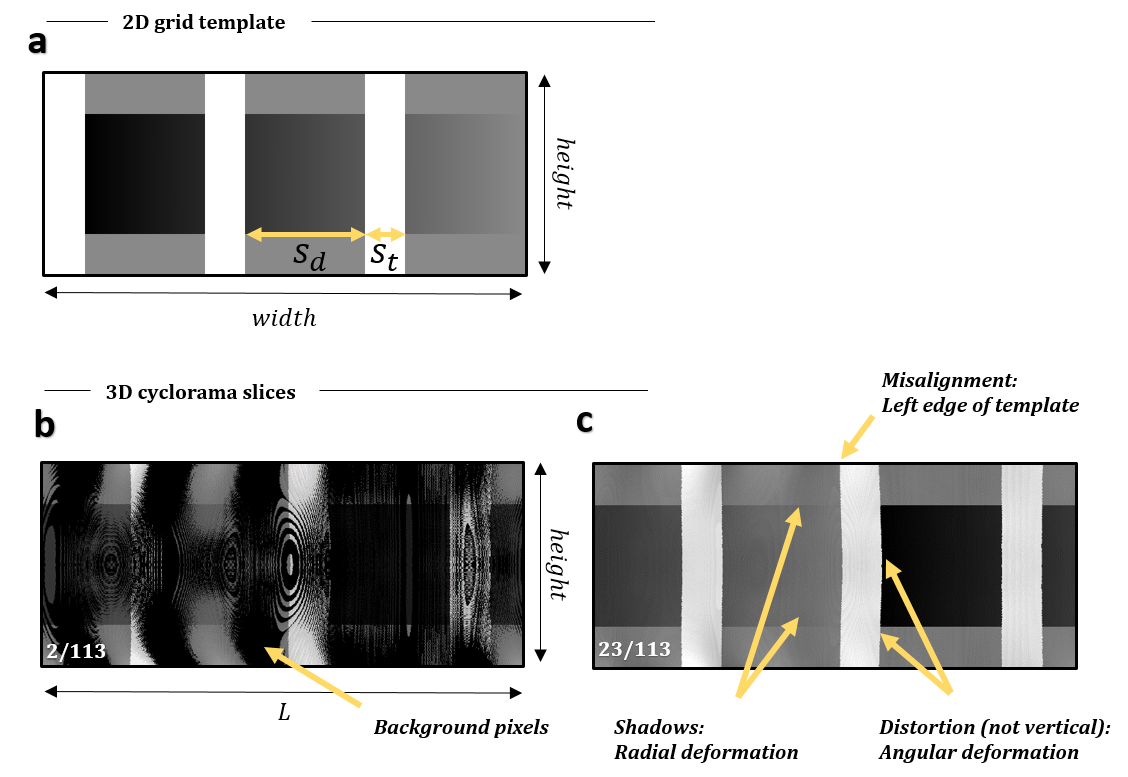

Supplement: Supplementary file 2 — Supplementary Figure S1 [file 41598_2021_93184_MOESM2_ESM.png]

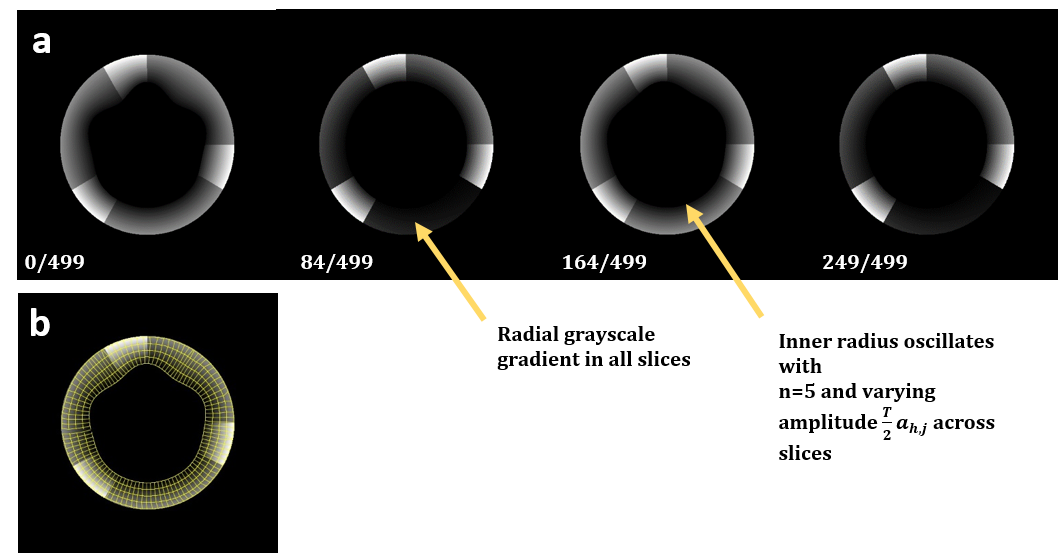

Supplement: Supplementary file 3 — Supplementary Figure S2 [file 41598_2021_93184_MOESM3_ESM.png]

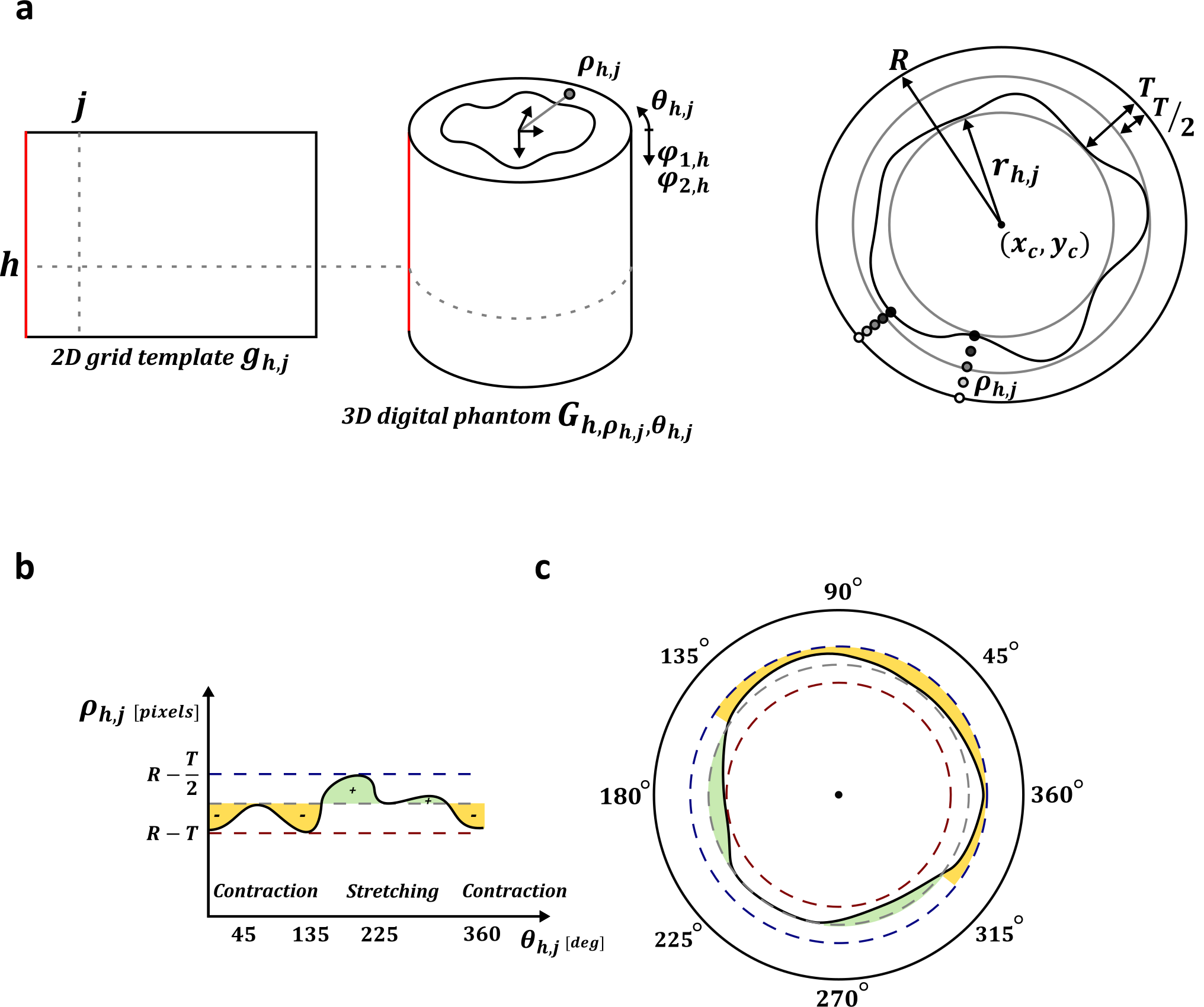

Supplement: Supplementary file 4 — Supplementary Figure S3 [file 41598_2021_93184_MOESM4_ESM.png]

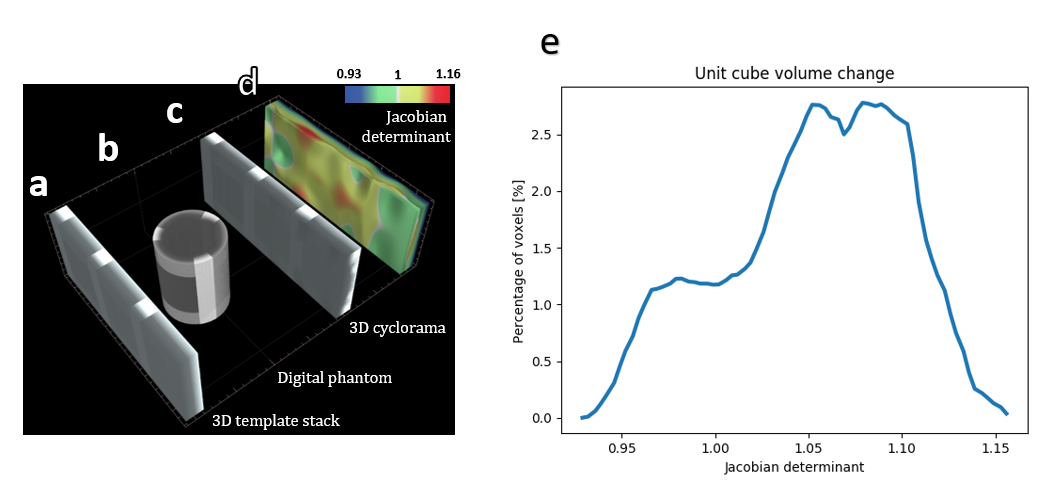

Supplement: Supplementary file 5 — Supplementary Figure S4 [file 41598_2021_93184_MOESM5_ESM.png]

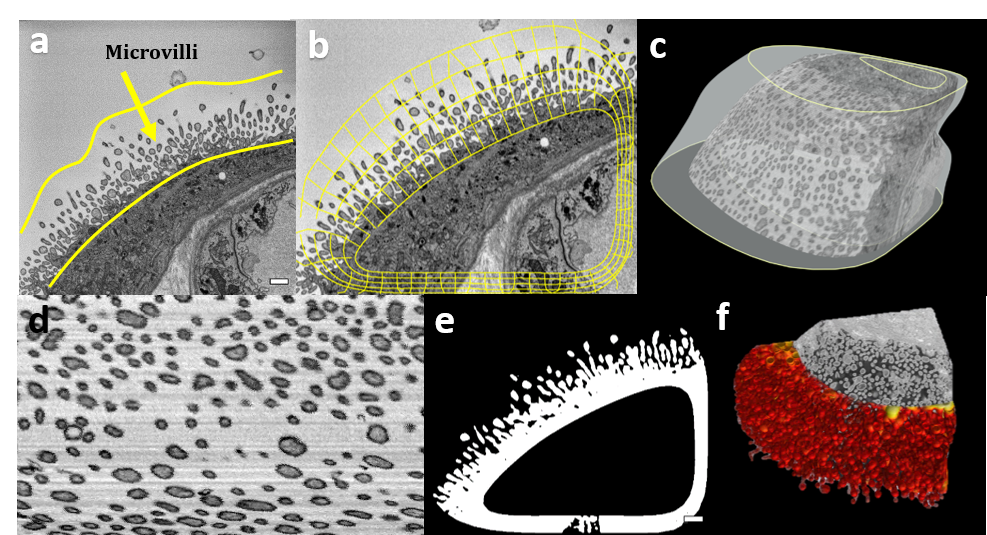

Supplement: Supplementary file 6 — Supplementary Figure S5 [file 41598_2021_93184_MOESM6_ESM.png]

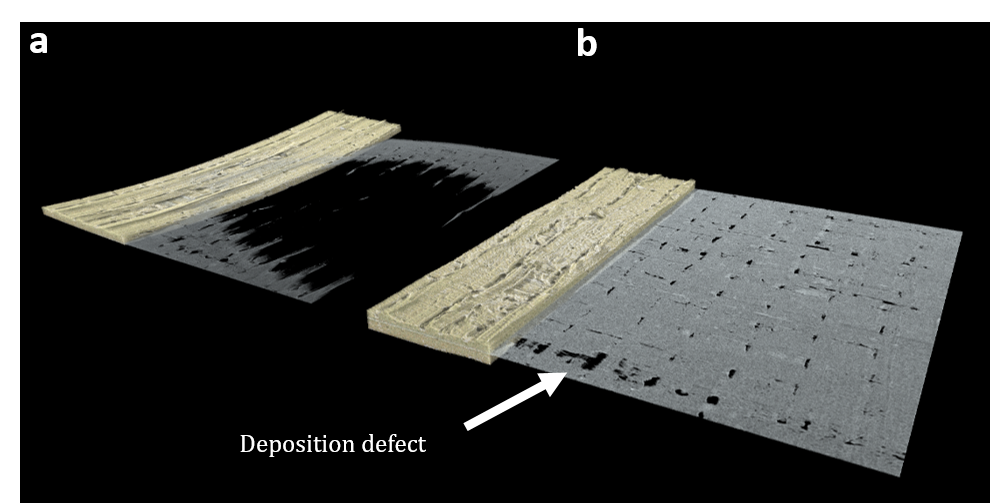

Supplement: Supplementary file 7 — Supplementary Figure S6 [file 41598_2021_93184_MOESM7_ESM.png]
